# Feasibility of Using the Video-Head Impulse Test to Detect the Involved Canal in Benign Paroxysmal Positional Vertigo Presenting With Positional Downbeat Nystagmus

**DOI:** 10.3389/fneur.2020.578588

**Published:** 2020-10-15

**Authors:** Andrea Castellucci, Pasquale Malara, Salvatore Martellucci, Cecilia Botti, Silvia Delmonte, Silvia Quaglieri, Elisabetta Rebecchi, Enrico Armato, Massimo Ralli, Marco Lucio Manfrin, Angelo Ghidini, Giacinto Asprella Libonati

**Affiliations:** ^1^ENT Unit, Department of Surgery, Azienda USL - IRCCS di Reggio Emilia, Reggio Emilia, Italy; ^2^Audiology and Vestibology Service, “Centromedico Bellinzona”, Bellinzona, Switzerland; ^3^ENT Unit, “Santa Maria Goretti” Hospital, Azienda USL Latina, Latina, Italy; ^4^PhD Program in Clinical and Experimental Medicine, University of Modena and Reggio Emilia, Modena, Italy; ^5^ENT Unit, Policlinico San Matteo Fondazione (IRCCS), Pavia, Italy; ^6^ENT Unit, “Guglielmo da Saliceto” Hospital, Piacenza, Italy; ^7^ENT Unit, “SS Giovanni e Paolo” Hospital, Venice, Italy; ^8^Head and Neck Department, ENT Clinic, Policlinico Umberto I, Rome, Italy; ^9^Department of Sense Organs, Sapienza University of Rome, Rome, Italy; ^10^Vestibology and ENT Unit, “Giovanni Paolo II” Hospital, Matera, Italy

**Keywords:** benign paroxysmal positional vertigo (BPPV), downbeat nystagmus, positional nystagmus, video head impulse test (vHIT), vestibulo-ocular reflex (VOR), poserior semicircular canal BPPV, anterior semicircular canal BPPV, apogeotropic BPPV

## Abstract

Positional downbeat nystagmus (pDBN) represents a relatively frequent finding. Its possible peripheral origin has been widely ascertained. Nevertheless, distinguishing features of peripheral positional nystagmus, including latency, paroxysm and torsional components, may be missing, resulting in challenging differential diagnosis with central pDBN. Moreover, in case of benign paroxysmal positional vertigo (BPPV), detection of the affected canal may be challenging as involvement of the non-ampullary arm of posterior semicircular canal (PSC) results in the same oculomotor responses generated by contralateral anterior canal (ASC)-canalolithiasis. Recent acquisitions suggest that patients with persistent pDBN due to vertical canal-BPPV may exhibit impaired vestibulo-ocular reflex (VOR) for the involved canal on video-head impulse test (vHIT). Since canal hypofunction normalizes following proper canalith repositioning procedures (CRP), an incomplete canalith jam acting as a “low-pass filter” for the affected ampullary receptor has been hypothesized. This study aims to determine the sensitivity of vHIT in detecting canal involvement in patients presenting with pDBN due to vertical canal-BPPV. We retrospectively reviewed the clinical records of 59 consecutive subjects presenting with peripheral pDBN. All patients were tested with video-Frenzel examination and vHIT at presentation and after resolution of symptoms or transformation in typical BPPV-variant. BPPV involving non-ampullary tract of PSC was diagnosed in 78%, ASC-BPPV in 11.9% whereas in 6 cases the involved canal remained unidentified. Presenting VOR-gain values for the affected canal were greatly impaired in cases with persistent pDBN compared to subjects with paroxysmal/transitory nystagmus (*p* < 0.001). Each patient received CRP for BPPV involving the hypoactive canal or, in case of normal VOR-gain, the assumed affected canal. Each subject exhibiting VOR-gain reduction for the involved canal developed normalization of vHIT data after proper repositioning (*p* < 0.001), proving a close relationship with otoliths altering high-frequency cupular responses. According to our results, overall vHIT sensitivity in detecting the affected SC was 72.9%, increasing up to 88.6% when considering only cases with persistent pDBN where an incomplete canal plug is more likely to occur. vHIT should be routinely used in patients with pDBN as it may enable to localize otoconia within the labyrinth, providing further insights to the pathophysiology of peripheral pDBN.

## Introduction

Positional downbeat nystagmus (pDBN) represents one of the most common findings related to central nervous system (CNS) disorders involving brainstem and cerebellum. As the main function of central vestibular system is to estimate the angular velocity, gravity orientation, and inertia processing peripheral vestibular afferents within the velocity-storage circuit, any lesions disrupting this network can generate pDBN (1, 2). Though central pDBN may also present with paroxysmal course, purely vertical direction, long duration, lack of latency, fatigability and no suppression with visual fixation represent the most prominent features of pDBN of central origin (3–7). Nevertheless, it has been widely demonstrated how pDBN may not rarely occur also in peripheral pathologies (1–3, 8). It can be elicited when the patient is brought into the straight head hanging (SHH) position and/or by Dix Hallpike (DH) maneuvers and it has been mainly related to benign paroxysmal positional vertigo (BPPV) involving the anterior semicircular canal (ASC) (3, 9–15). Despite detached otoconia, moving inside unusual sites of the labyrinth, represent the assumed underlying mechanism, peripheral pDBN patterns show features classically known as central such as lack of torsional components and long time constant (9, 11, 15). More recently, it has been hypothesized that even otoliths settling in the distal portion of the non-ampullary tract of the posterior semicircular canal (PSC) may result in pDBN (16–27). This type of PSC-BPPV has been named “apogeotropic variant” (18, 20) as nystagmus evoked in provoking positions beats away from the ground and in the opposite direction to positional paroxysmal upbeat nystagmus (beating toward the ground in DH positioning, therefore geotropic) due to classical BPPV involving PSC ampullary arm. Demi-Semont (DS) maneuver, 45°-forced prolonged position (FPP) and quick liberatory rotation represent physical treatments proposed for this PSC-BPPV variant, with the aim of moving back displaced particles to the vestibule (19, 20). Nevertheless, it is not rarely hard to identify the affected semicircular canal (SC) due to the possible missing torsional components in pDBN (9, 11, 15, 19, 20). Additionally, ASC-BPPV is generally hardly distinguishable from contralateral apogeotropic variant of PSC-BPPV as in both cases resulting pDBN is generated by the contraction of the same ocular muscles (18, 28). Thanks to the introduction of video-head impulse test (vHIT) in clinical practice, high-frequency VOR measurements for semicircular canals can be easily assessed (29, 30). This new clinical device has been widely used to measure SC function, in both peripheral and central vestibular disorders (31–36). Recently, it has been assumed that vestibulo-ocular reflex (VOR) for the affected SC may be impaired in BPPV resulting in pDBN, providing possible key data for differential diagnosis (37). To further investigate this claim, we submitted a homogeneous cohort of patients with pDBN due to vertical SC-BPPV to statistical analysis and assessed the diagnostic sensitivity of vHIT in detecting the SC involved by BPPV among cases presenting with pDBN. Reviewing our results, we also aimed to offer possible explanations for VOR-gain abnormalities for the affected SC in such cases, providing better insights to the pathophysiology of peripheral pDBN.

## Materials and Methods

### Patients

This study was approved by our Institutional Review Boards (approval number for the promoter center: 236/2020/OSS/AUSLRE) and was conducted according to the tenets of the Declaration of Helsinki. We performed a retrospective review of clinical-instrumental data of a cohort of 93 patients presenting with pDBN who were evaluated at our centers between June 2019 and May 2020. Overall subjects were admitted either to the outpatient units or to the emergency units. In order to select only patients with peripheral pDBN due to vertical SC-BPPV, subjects exhibiting oculomotor central signs (gaze-evoked nystagmus, rebound nystagmus, pDBN not reduced or enhanced by visual fixation) or abnormal findings on gadolinium-enhanced magnetic resonance imaging (MRI) were excluded from the analysis. Likewise, patients with past history of vestibular pathologies potentially resulting in pDBN or possible VOR-gain abnormalities for vertical SCs [i.e., Meniere's disease (38), vestibular migraine (39), inferior vestibular neuritis (40), sudden sensorineural hearing loss with vertigo (33, 41), canal dehiscences (42)], were excluded. Therefore, only patients with pDBN receding or converting into a typical BPPV positional nystagmus following proper canalith repositioning procedures (CRP) were considered. Among Authors, AC, PM, SM, SQ, ER, and EA (all neurotologists) were directly involved in the analysis of pDBN features and data collection. Patients without complete clinical data including at least pre- and post-treatment measurements for all six SC VOR-gains on vHIT were not included in the study. Finally, a residual homogeneous population of 54 patients was recruited for statistical analysis. All patients underwent the same detailed work-up including history taking and bedside examination with the aid of video-Frenzel goggles or video-oculography (VOG). Each patient underwent a comprehensive assessment for all SCs VOR-gains on vHIT before and after physical treatment and only few of them were submitted to VEMPs in different stages of BPPV. Gadolinium-enhanced MRI and/or temporal bones high-resolution CT (HRCT) scan were performed if needed. Besides personal details, patients were asked whether recent head trauma occurred. They were also investigated for history of BPPV with paroxysmal positional nystagmus documented by Video-Frenzel goggles within 30 days prior to examination. Additionally, patients were divided into subgroups according both to the time elapsed between symptoms onset and clinical assessment (<7 and >7 days) and to days needed for pDBN either to recede or to convert in typical positional nystagmus due to typical ipsilateral canalolithiasis (<7 and >7 days).

### Detection of the Vertical Canal Affected by BPPV

Any of the following strategies were used for the identification of the SC involved by BPPV:

- Detection of the SC with impaired VOR-gain values on vHIT with either covert or overt saccades.
- In case of pDBN with torsional components, recent history of BPPV with paroxysmal positional nystagmus documented with Video-Frenzel goggles addressed the diagnosis toward a specific SC (i.e., recent left PSC-BPPV addressed the diagnosis toward ipsilateral ASC-BPPV in a patient presenting with pDBN with leftbeating torsional nystagmus, whereas rightbeating components would reasonably indicate ipsilateral apogeotropic PSC-BPPV).

In cases lacking of the above-mentioned findings, detection of the affected canal could only be provided after physical treatment, basing on the following findings:

- Resolution of pDBN after proper CRP designed to release a specific SC from debris, as therapeutic maneuvers, though effective in moving debris, would not result in restoring BPPV affecting other SCs.
- Conversion of pDBN in classical paroxysmal positional nystagmus involving either ipsilateral PSC (upbeating/torsional nystagmus on ipsilateral DH positioning) or horizontal SC (HSC) (either geotropic or apogeotropic horizontal direction-changing nystagmus at the supine head roll test) after any CRP performed, consistently with otoconial switch from the affected canal either to other ipsilateral SCs or to another tract of the same affected SC (i.e., conversion of pDBN with leftbeating torsional components into paroxysmal upbeating nystagmus with rightbeating torsional components elicited in right positioning consistently with debris shift into right PSC ampullary arm addressed the original diagnosis toward ipsilateral BPPV involving PSC non-ampullary arm rather than contralateral ASC-BPPV).

### Physical Treatment

All patients underwent specific physical therapy aimed to move debris back to the utricle from the assumed affected vertical SC. In cases with BPPV involving the non-ampullary tract of PSC, DS maneuver was mainly used, followed by the 45°-FPP in case of persistence of symptoms following DS (20). Whereas DS maneuver mainly exploits inertial force to free the affected SC from otoconia, as it basically represents the second part of the well-known Semont's liberatory maneuver (43), 45°-FPP technique uses gravity to move particles toward the utricle, as the affected PSC is located in the uppermost part of the labyrinth in this position (20). Standard Epley's CRP (44) or Semont's maneuver were rarely used as first therapeutic choice, mainly depending on examiner's preferences or patient's compliance.

In cases with ASC involvement, patients were mostly treated with Yacovino's technique (45), followed by prolonged forced position procedure (PFPP) (46) in subjects not exhibiting immediate recovery.

In cases where affected SC could not be ascertained due to the lack of the aforementioned findings, several CRP were pursued according to examiner's experience to obtain a canal switch, to move otoconia toward another tract of the involved SC or to directly free the affected canal.

Each subject was checked within 3–4 days. In case of persistence of pDBN, additional CRP were pursued with following check within further 3–4 days, and so on until a complete recovery or a canal switch was achieved. Physical therapy outcome was considered as successful either if patients were free from symptom and signs or if they exhibited a conversion into a typical form of BPPV. In case debris moved to the ampullary tract of PSC, Epley's or Semont's maneuvers were performed according to examiner's preference or patient's compliance, whereas proper CRP for geotropic and apogeotropic variants of HSC-BPPV were used (47, 48) in case debris moved either to non-ampullary or to ampullary arm of HSC, respectively. All patients were finally checked within further 3–4 days for ensuring a complete recovery.

### Eye Movements Recording

Eye movements were analyzed with video-Frenzel goggles or video-oculography (VOG). Horizontal, vertical and torsional nystagmus were qualitatively assessed. Horizontal (right/leftbeating), vertical (up/downbeating) directions of nystagmus and torsional components (right/leftbeating, i.e., with the upper pole of the eye rotating toward the right/left ear, respectively) were described from the patient's point of view. Bedside-examination included assessment of spontaneous and positional nystagmus evoked by both DH and SHH positionings. Once evaluated any spontaneous DBN (purely vertical, with or without torsional components), positional nystagmus was checked for latency (with/without), direction (purely vertical or with torsional components), inhibition with visual fixation (yes/no), duration and temporal trend (either transitory/paroxysmal accompanied by a crescendo-decrescendo pattern if <2 min, or persistent with nearly stationary course if >2 min) and reversal when returning upright following positionings (with/without).

### vHIT

Vestibulo-ocular reflex (VOR) gains for all three SCs were tested on both sides in response to high-frequency head stimuli on vHIT, an ICS video-oculographic system (GN Otometrics, Denmark). At least 15 impulses were delivered for stimulating each SC and averaged to get corresponding mean VOR-gains. Vertical SC were considered hypoactive if VOR-gains were <0.7 with at least either covert or overt saccades (29, 30). All patients underwent vHIT testing at the presentation and following CRP, whether they succeed in SC releasing or resulted in a conversion into a typical BPPV variant (i.e., as soon as pDBN either receded or converted in positional paroxysmal nystagmus).

### VEMPs Testing

Cervical and ocular vestibular-evoked myogenic potentials (cVEMPs and oVEMPs, respectively) for air-conducted sounds were recorded using 2-channel evoked potential acquisition systems (either Neuro-Audio, Neurosoft, Russia or Viking, Nicolet EDX, CareFusion, Germany depending on different centers) with surface electrodes placed according to standardized criteria (49). Potentials were recorded delivering tone bursts (frequency: 500 Hz, duration: 8 ms, stimulation rate: 5 Hz) via headphones either before or following CRP. Recording system used an EMG-based biofeedback monitoring method to minimize variations in muscles contractions and VEMPs amplitudes. A re-test was performed for each stimulus to assess reproducibility. The first biphasic responses on the ipsilateral sternocleidomastoid muscle (p13-n23) for cVEMPs (ipsilateral response) and under the patient's contralateral eye (n10-p15) for oVEMPs (crossed response) were analyzed by calculating the peak-to-peak amplitude. Inter-aural amplitude difference between ear affected (Aa) and unaffected (Au) by BPPV were calculated with the asymmetry-ratio (AR): [(Au – Aa)/(Au + Aa)] × 100. Otolith sensors on the pathologic side were considered damaged if potentials resulted in AR >35%, according to our normative data and to literature references (49).

### Statistical Analysis

Quantitative variables were checked for normal distribution using both Kolmogorov-Smirnov and Shapiro-Wilk tests. Continuous variables were described by mean ± 1 standard deviation for normally distributed variables or by median, interquartile range and range for non-normally distributed variables. Diagnostic sensitivity of vHIT in detecting the involved SC in BPPV with pDBN was calculated as the ratio of cases with hypoactive SC to overall patients. Conversely, diagnostic sensitivity of vHIT for persistent pDBN was calculated as the ratio of cases with persistent pDBN exhibiting a deficient SC to overall cases with persistent pDBN, whereas vHIT sensitivity for transitory/paroxysmal pDBN was derived dividing the number of cases with transitory/paroxysmal pDBN presenting with a hypoactive SC for overall cases exhibiting transitory/paroxysmal pDBN. Fisher's exact test was used for categorical comparisons. Spearman's rank correlation was used to correlate patient's age with SCs VOR-gains. Wilcoxon signed-rank test was used to compare pre- and post-treatment vHIT data for all six SCs. Mann-Whitney *U*-test was employed for pairwise comparisons between subgroups. Results were considered statistically significant if *p* < 0.05. Statistical analyses were performed using IBM SPSS ver. 20.0 (IBM Corp., Armonk, NY, USA).

## Results

Fifty-four patients (20 males, 34 females, mean age 55.9 ± 13.8) with pDBN due to vertical SC-BPPV were included in the study. Recurrence of pDBN due to BPPV involving the same SC was recorded in a case and it was considered twice in the analysis. Similarly, four patients (1 male and 3 females) were considered twice as they exhibited either simultaneous or newly sequential BPPV involving other vertical SCs with pDBN. Therefore, clinical and instrumental data concerning 59 cases with pDBN due to vertical SC-BPPV were finally analyzed. Detailed information about overall 59 cases included in the study can be found in Supplementary Tables 1, 2.

**Figure 1:**
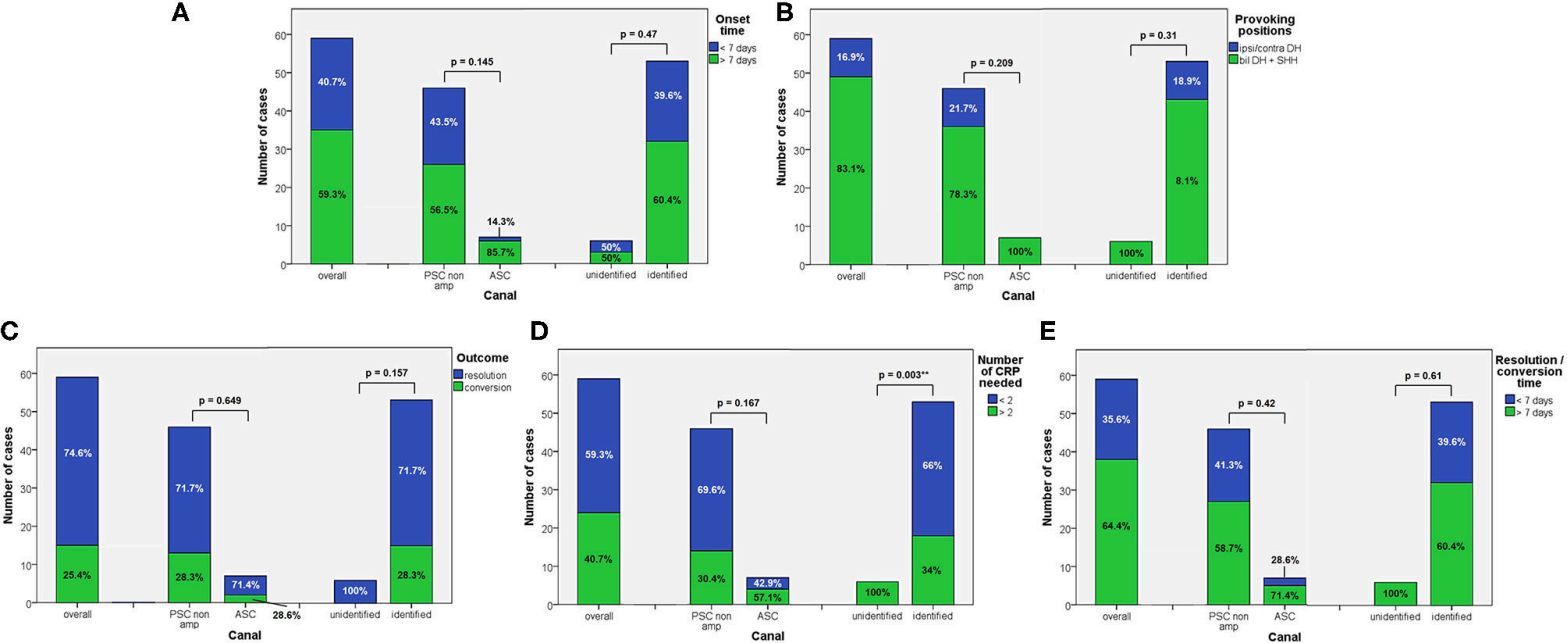
Bar plots showing the amount of subjects among overall population of 59 cases, divided in paired subgroups based on diagnosis, exhibiting different clinical features. **(A)** Time of symptoms onset. **(B)** Provoking positions. **(C)** Outcome. **(D)** Number of CRP needed. **(E)** Resolution or conversion time. Relative percentages among subgroups are reported in each column. Statistically significant differences at the Fisher's exact test are reported and highlighted with **p < 0.01, respectively. ASC, anterior semicircular canal; CRP, canalith repositioning maneuvers; PSC non-amp, posterior semicircular canal non-ampullary arm.

**Figure 2:**
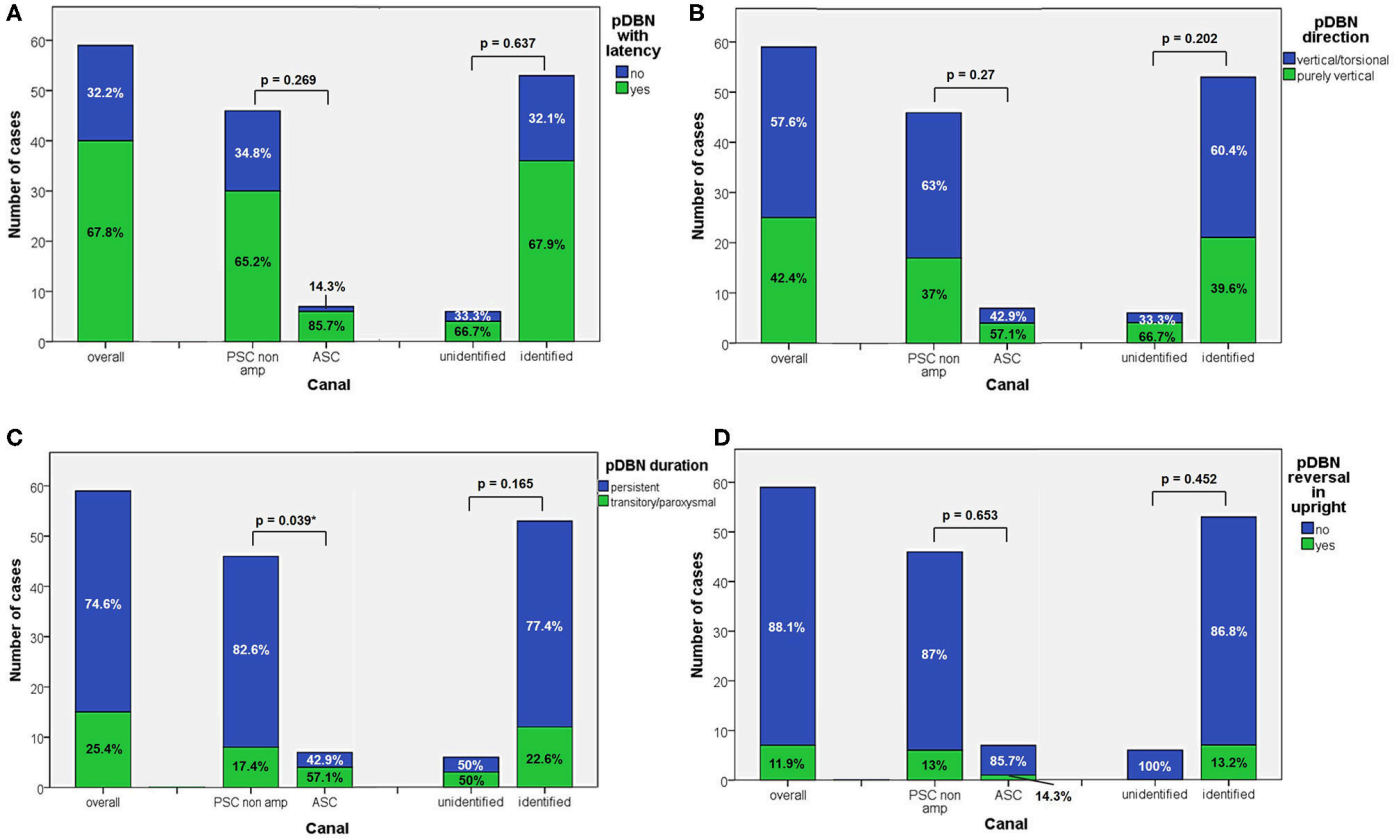
Bar plots showing the amount of subjects among overall population of 59 cases, divided in paired subgroups based on diagnosis, presenting with different VOG features. **(A)** pDBN with latency. **(B)** pDBN direction. **(C)** pDBN duration. **(D)** pDBN reversal in upright. Relative percentages among subgroups are reported in each column. Statistically significant differences at the Fisher's exact test are reported and highlighted with *p < 0.05, respectively. ASC, anterior semicircular canal; pDBN, positional downbeat nystagmus; PSC non-amp, posterior semicircular canal non-ampullary arm; VOG, video-oculography.

Apogeotropic PSC-BPPV was diagnosed in 78% of cases (46/59, 26 on right and 20 on left side), ASC-BPPV in 11.9% of cases (7/59, all left-sided) whereas in 6 cases (10.1%) neither the involved SC nor the pathologic side could be ascertained (Table 1). Recent BPPV were reported in 46 cases (77.9%) and in 10 cases (16.9%) previous head trauma could be recorded (Table 1). Most subjects (35/59, 59.3%) presented at our attention more than a week from symptoms onset, without statistically significant difference between apogeotropic PSC-BPPV and ASC-BPPV cases or between subjects with identified SC and unknown affected site (Figure 1A). In patients with BPPV involving PSC non-ampullary arm, spontaneous purely vertical DBN was identified in 2 subjects and spontaneous torsional/vertical DBN was identified in 4 cases, consistently with a canalith jam. Spontaneous nystagmus did not exhibit direction changings either in forward or backward head bending along the pitch plane, was inhibited by visual fixation in its vertical component and increased in recumbent positionings in all 6 subjects. It likely resulted from previously performed CRP only in half of cases, whereas the remaining 3 patients presented with a spontaneous canalith jam converting into an ipsilateral SC-BPPV after DS maneuvers with the aid of mastoid vibrations. While 83.1% of overall cases and the whole population either with ASC-BPPV or BPPV involving undefined SC presented with pDBN detectable in both SHH and bilateral DH positions, both maneuvers resulted in positional nystagmus only in 78.3% of cases with apogeotropic PSC-BPPV, without statistically significant difference among subgroups (Figure 1B). Overall rate of pDBN presenting with latency was 67.8% (40/59) with similar ratios in different BPPV-subtypes (65.2% in apogeotropic PSC-BPPV, 85.7% in ASC-BPPV and 67.8% in cases with unidentified SC) (Figure 2A). Thirty-four patients (57.6%) exhibited positional nystagmus with torsional components, whereas pDBN direction was purely vertical in 25 cases (42.4%) without significant differences among subgroups. Nevertheless, the majority of patients with BPPV involving either ASC (57.1%) or undefined SC (66.7%) presented pDBN lacking of torsional components (Figure 2B). In most cases (44/59, 74.6%) pDBN was persistent in provoking positions showing significant higher prevalence among subjects with debris settling the non-ampullary tract of PSC (*p* = 0.039) compared to ASC involvement. Conversely, it exhibited transitory and typical paroxysmal crescendo-decrescendo course in most cases with ASC-BPPV (4/7, 57.1%) and in half of patients where the affected SC was not identified (Figure 2C). The vast majority of cases (52/59, 88.1%), irrespective to the involved SC, lacked in reversal of positional nystagmus once returning in upright position, being totally absent in cases with no detectable pathologic site (Figure 2D). No significant difference in terms of outcome with physical therapy could be found among underlying diagnosis, being resolution of pDBN predominant over conversion into typical paroxysmal nystagmus due to either canal switch or progression toward the ampullary tract of PSC in all different BPPV-subtypes (Figure 1C). Despite two types of CRP (usually an impulsive maneuver followed by prolonged positioning) were enough either to release the involved canal from dislodged particles or to convert pDBN into paroxysmal nystagmus in most cases (35/59, 59.3%), all subjects with unidentified affected SC received more than 2 types of CRP to recover (*p* = 0.003; Figure 1D). Conversely, no substantial difference in terms of time needed to recover or convert into typical BPPV could be found among different subgroups, prevailing a time-period greater than a week across all different BPPV forms (Figure 1E).

**Table 1:** Information about personal details, history, and VOR-gain abnormalities for overall 59 cases with pDBN and different subgroups divided according to the vertical semicircular canal involved by BPPV.

**Affected semicircular canal (% of overall)**	**Sex**		**Age (y)**	**Previous BPPV**		**Previous head trauma (%)**	**VOR-gain abnormalities (%)**
	**M (%)**	**F (%)**		**Defined (%)**	**Undefined (%)**		
Overall, *n*. 59 (100)	21 (35.6)	38 (64.4)	57 ± 13.9	33 (55.9)	13 (22)	10 (16.9)	43 (72.9)
PSC, *n*. 46 (78)	16 (34.8)	30 (65.2)	57.9 ± 14.4	26 (56.5)	10 (21.7)	6 (13)	37 (80.4)
ASC, *n*. 7 (11.9)	2 (28.6)	5 (71.4)	59.7 ± 11.8	6 (85.7)	0 (0)	2 (28.6)	6 (85.7)
Unidentified, *n*. 6 (10.1)	3 (50)	3 (50)	47.3 ± 8.2	1 (16.7)	3 (50%)	2 (33.3)	0 (0)

*ASC, anterior semicircular canal; BPPV, benign paroxysmal positional vertigo; F, female; M, male; pDBN, positional downbeat nystagmus; PSC, posterior semicircular canal; VOR, vestibulo-ocular reflex; y, years*.

**Figure 3:**
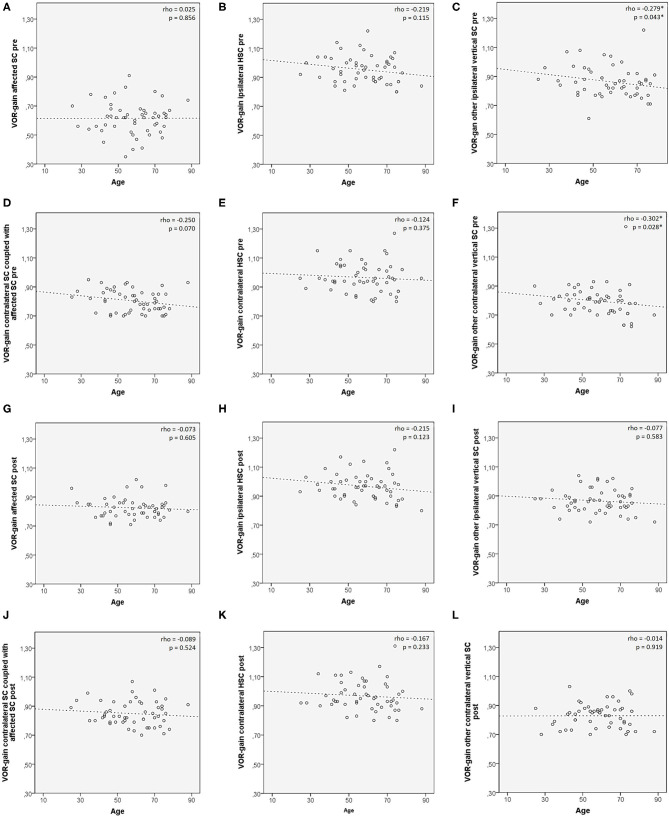
Scatter plots correlating patients' age among 53 patients with identified affected SC with presenting VOR-gain **(A–F)** and post-treatment VOR-gain **(G–L)** for each semicircular canal. Each plot shows the linear regression line with corresponding Spearman's correlation coefficient (ρ). *p* < 0.05 are reported and marked with *. HSC, horizontal semicircular canal; post, post-treatment; pre, at presentation; SC, semicircular canal; VOR, vestibule-ocular reflex.

Preliminary correlation analysis between patient's age and VOR-gain values for each SC (both pre- and post-treatment) in subjects with defined affected SC (*n*. 53) was performed prior to investigating VOR-gain behavior among different subgroups, to ensure lack of consistent age-related bias involving canal activity (50). Only a negative correlation between patients' age and presenting VOR-gains for the other vertical SC ipsilaterally to the affected canal (rho = −0.279, *p* = 0.043) and for the contralateral SC other than the canal coupled with the affected SC (rho = −0.302, *p* = 0.028) was found (Figure 3).

**Figure 4:**
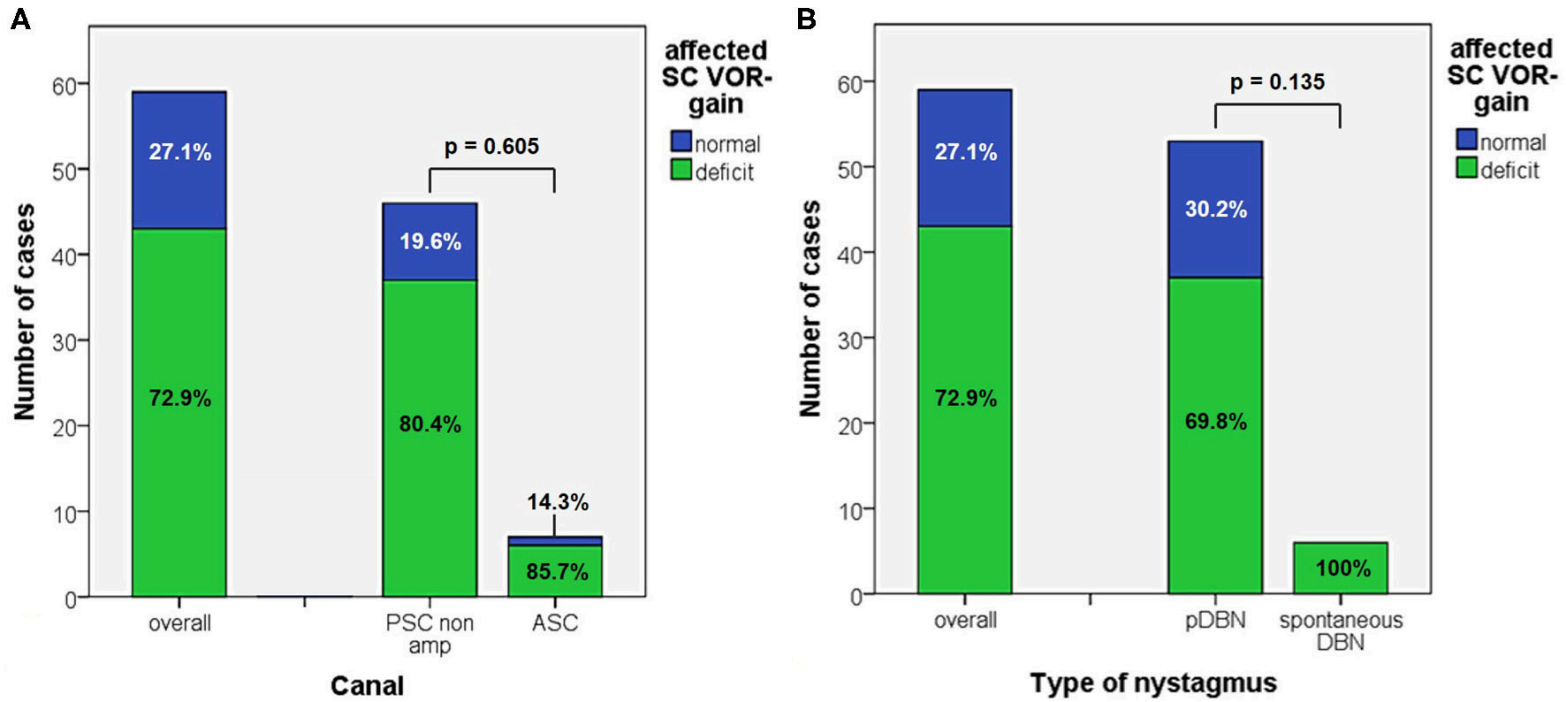
Bar plots showing the amount of patients, divided in paired subgroups based on diagnosis **(A)** or on type of positional nystagmus **(B)**, exhibiting normal or hypoactive VOR-gain for the affected semicircular canal at presentation. Relative percentages among subgroups are reported in each column. Statistically significant differences at the Fisher's exact test are reported. ASC, anterior semicircular canal; pDBN, positional downbeat nystagmus; PSC non-amp, posterior semicircular canal non-ampullary arm; SC, semicircular canal; VOR, vestibulo-ocular reflex.

**Figure 5:**
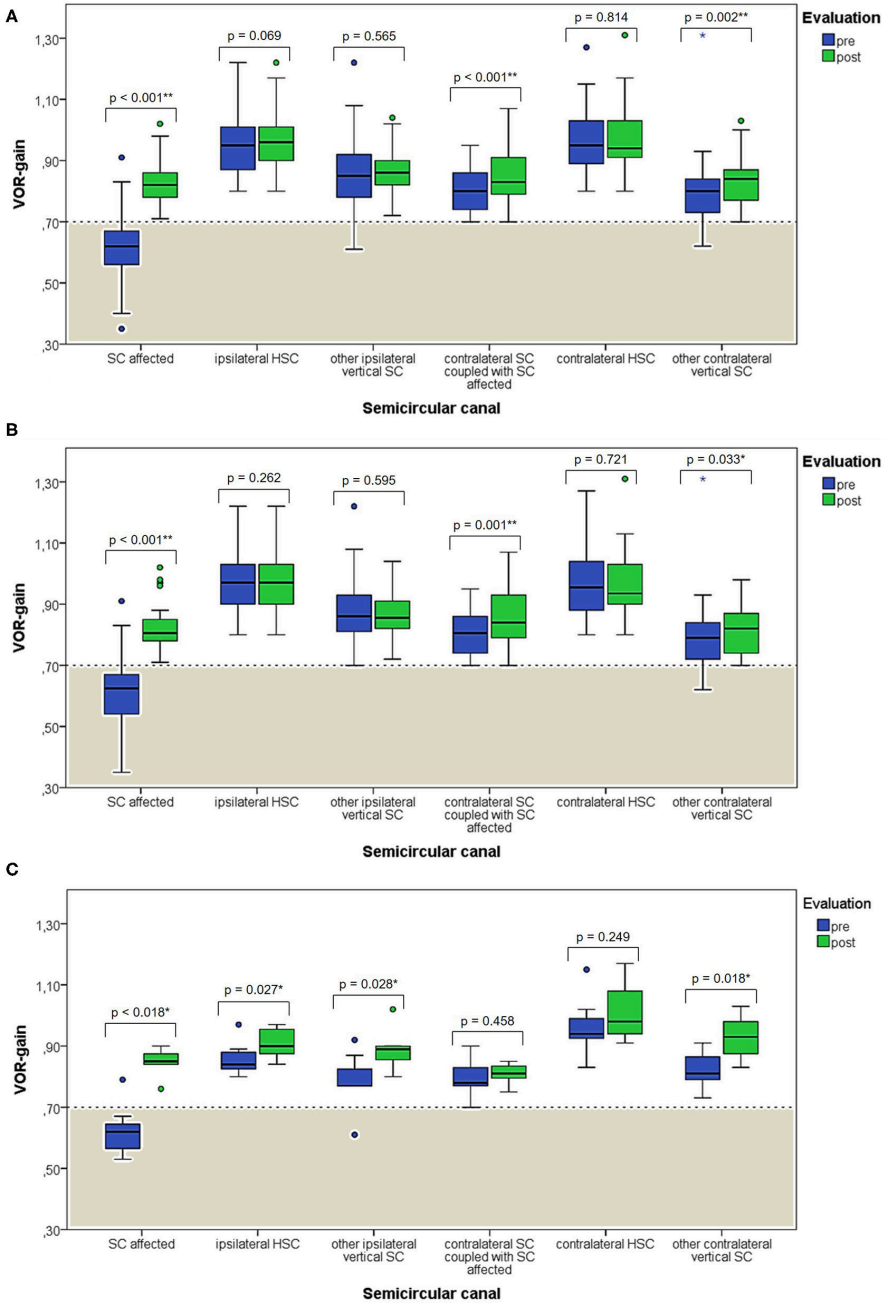
Box plots correlating medians of VOR-gain values at presentation and following physical therapy for each semicircular canal among overall 53 subjects with identified affected SC **(A)**. The same correlation is shown considering either only cases with apogeotropic PSC-BPPV **(B)** or ASC-BPPV **(C)**. Horizontal dashed lines represent the border between normal and pathologic VOR-gain values for vertical canals (0.7) and values within gray areas represent abnormal measurements. Statistically significant differences at the Wilcoxon signed-rank test are shown. *p* < 0.05 and < 0.01 are marked with * and **, respectively. ASC, anterior semicircular canal; BPPV, benign paroxysmal positional vertigo; HSC, horizontal semicircular canal; post, post-treatment; pre, at presentation; PSC, posterior semicircular canal; SC, semicircular canal; VOR, vestibulo-ocular reflex. Values at a greater distance from the median than 1.5 times and 3 times the IQR are plotted individually as dots (weak outliers) and asterisks (strong outliers), respectively.

In 43/59 cases (72.9%) an isolated vertical SC hypofunction could be identified without statistically significant difference between apogeotropic PSC (37/46, 80.4%) and ASC-BPPV (6/7, 85.7%) (Figure 4A and Table 1). In all these patients, torsional components of pDBN, when detected, were in agreement with the excitatory (in ASC-BPPV) or inhibitory (in apogeotropic PSC-BPPV) discharge of the hypoactive SC, as expected from resulting endolymphatic flows elicited by otoconial shift in DH and SHH positionings. Moreover, proper CRP for treating the hypoactive SC succeeded either in resolution of symptoms and signs or in conversion into a typical ipsilateral BPPV with paroxysmal nystagmus involving the ampullary tract of PSC or HSC in all cases. All these patients exhibited normalization of VOR-gain abnormalities after either pDBN resolution or conversion into paroxysmal positional nystagmus, confirming a close linkage between transient high-frequency VOR impairment and BPPV-related pDBN (Figure 5). In none of our cases, deficient VOR-gain values could be detected for a SC unrelated to BPPV, except for a case who presented with impaired PSC VOR-gain ipsilaterally to the affected ASC (with normal VOR-gain) normalizing after successful physical therapy for ASC-BPPV. All patients presenting with spontaneous DBN exhibited hypoactive VOR-gain for the affected canal (Figure 4B).

**Figure 6:**
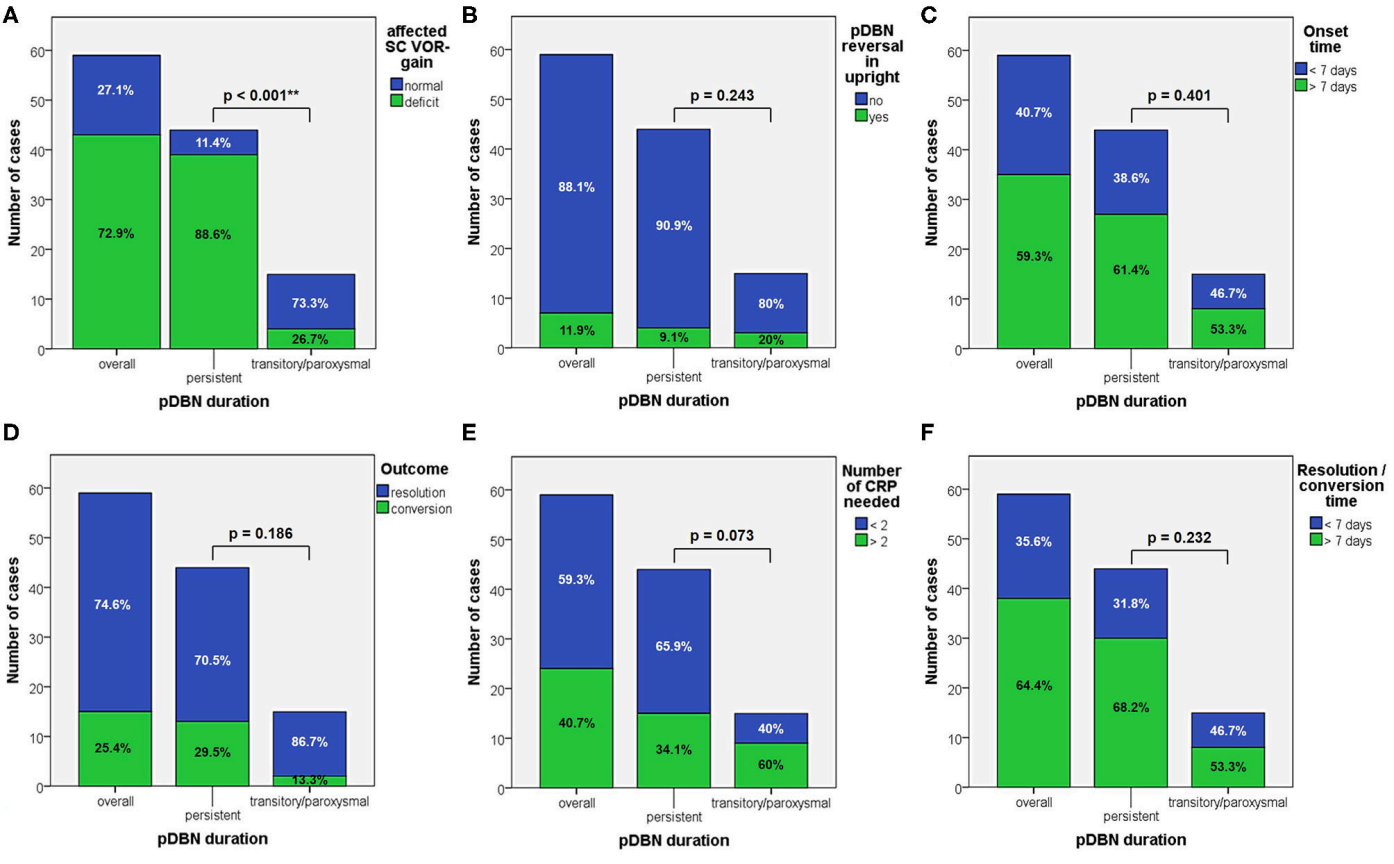
Bar plots showing the amount of subjects among overall population, divided in paired subgroups depending on pDBN duration, exhibiting different instrumental, and clinical features. **(A)** Normal or hypoactive SC VOR-gain for the affected canal at presentation. **(B)** Reversal of pDBN in upright position. **(C)** Different onset times. **(D)** Different outcomes. **(E)** Different number of CRP needed for resolution or conversion. **(F)** Different time needed for resolution or conversion of pDBN. Relative percentages among subgroups are reported in each column. Statistically significant differences at the Fisher's exact test are reported. CRP: canalith repositioning procedure. pDBN, positional downbeat nystagmus; SC, semicircular canal; VOR, vestibulo-ocular reflex. Statistically significant differences at the Fisher's exact test are reported and highlighted with **p < 0.01.

When dividing overall cohort of patients according to pDBN duration, subgroup of patients presenting with persistent positional nystagmus (44/59) exhibited a significantly higher rate of cases with VOR-gain impairment (88.6%) compared to patients in which positionings elicited a transient/paroxysmal pDBN (26.7%) (*p* < 0.001; Figure 6A). Nevertheless, rates of cases showing pDBN reversal in upright position, different time from symptoms onset and for recovery or conversion of pDBN, different outcomes and number of CRP required did not differ between subgroups exhibiting these two different pDBN patterns (Figures 6B–F).

**Figure 7:**
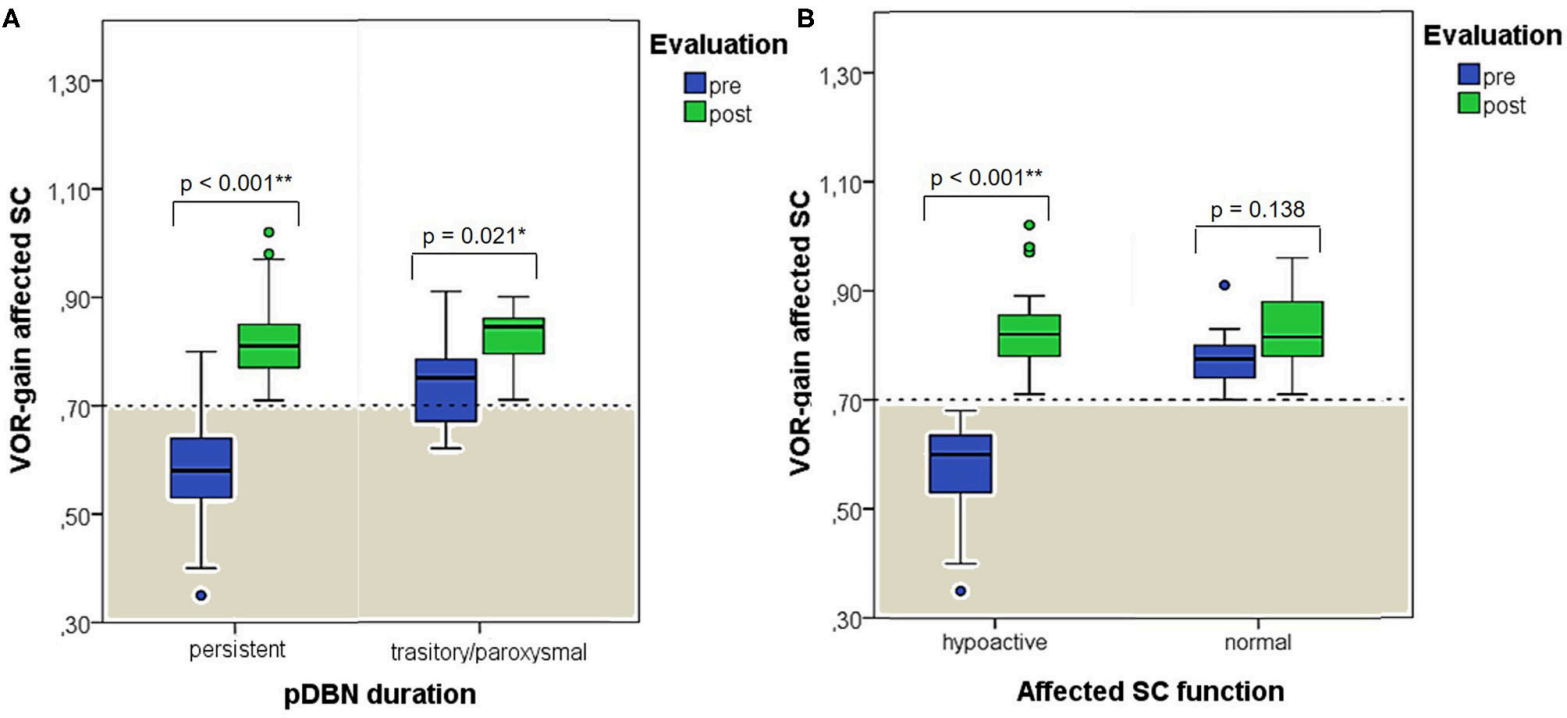
Box plots correlating medians of VOR-gain values at presentation and following physical therapy for the affected SC among overall 53 subjects with identified pathologic canal divided according to pDBN duration **(A)** and affected SC function **(B)**. Horizontal dashed lines represent the border between normal and pathologic VOR-gain values for vertical canals (0.7) and values within gray areas represent abnormal measurements. Statistically significant differences at the Wilcoxon signed-rank test are shown. *p* < 0.05 and < 0.01 are marked with * and **, respectively. pDBN, positional downbeat nystagmus; post, post-treatment; pre, at presentation; SC, semicircular canal; VOR, vestibulo-ocular reflex. Values at a greater distance from the median than 1.5 times the IQR are plotted individually as dots (weak outliers).

**Figure 8:**
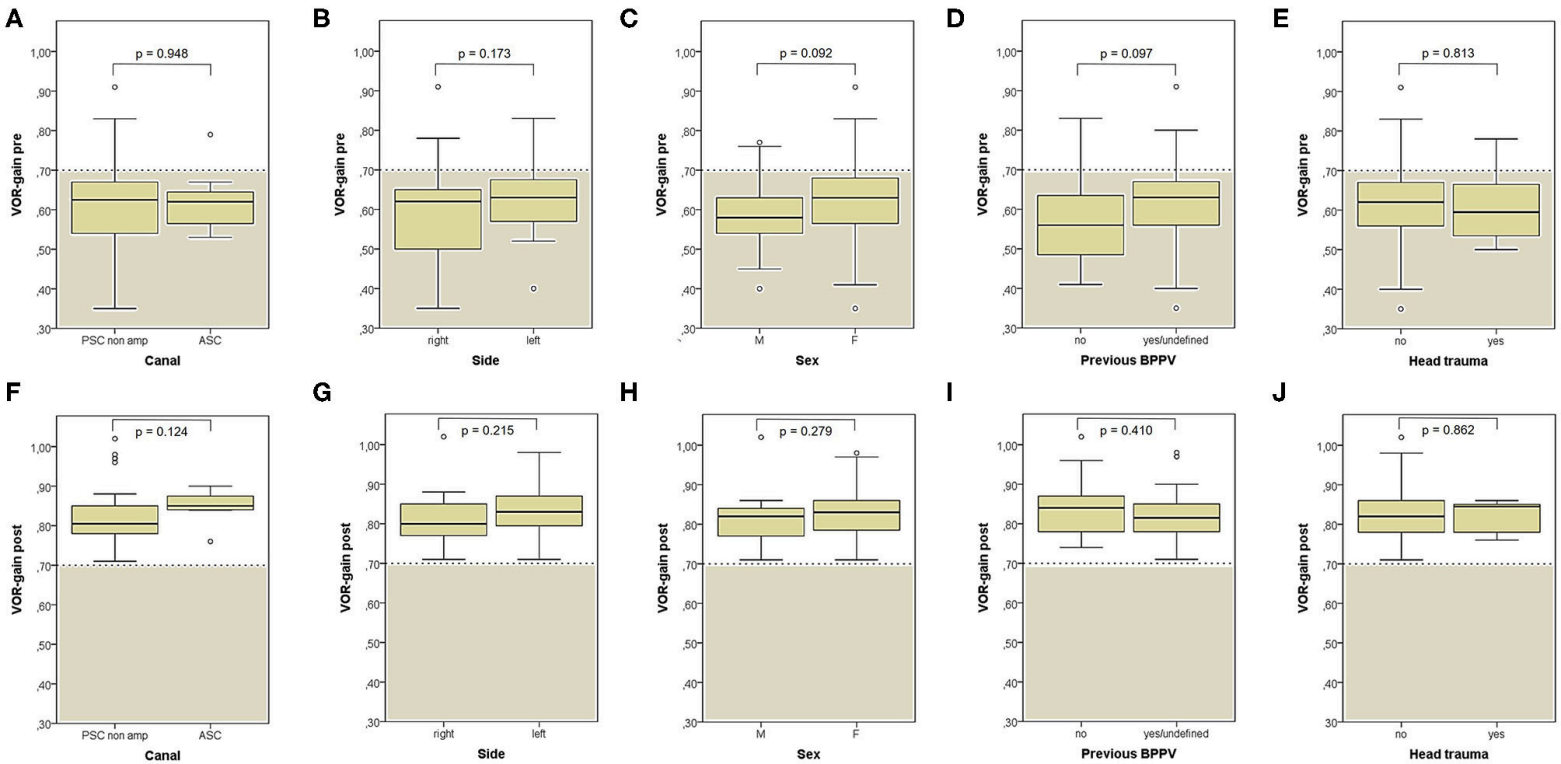
Box plots correlating medians of VOR-gain values at presentation **(A–E)** and following physical treatment **(F–J)** for the SC involved by BPPV among overall subjects (53) with identified affected canal divided based on different details. **(A,F)** Affected semicircular canal. **(B,G)** Affected side. **(C,H)** Gender. **(D,I)** History of recent BPPV. **(E,J)** History of recent head trauma. Statistically significant differences at the Mann-Whitney *U*-test are reported. ASC, anterior semicircular canal; BPPV, benign paroxysmal positional vertigo; F, female; M, male; post, post-treatment; pre, at presentation; PSC non-amp, posterior semicircular canal non ampullary arm; SC, semicircular canal; VOR, vestibulo-ocular reflex. Values at a greater distance from the median than 1.5 times the IQR are plotted individually as dots (weak outliers).

**Figure 9:**
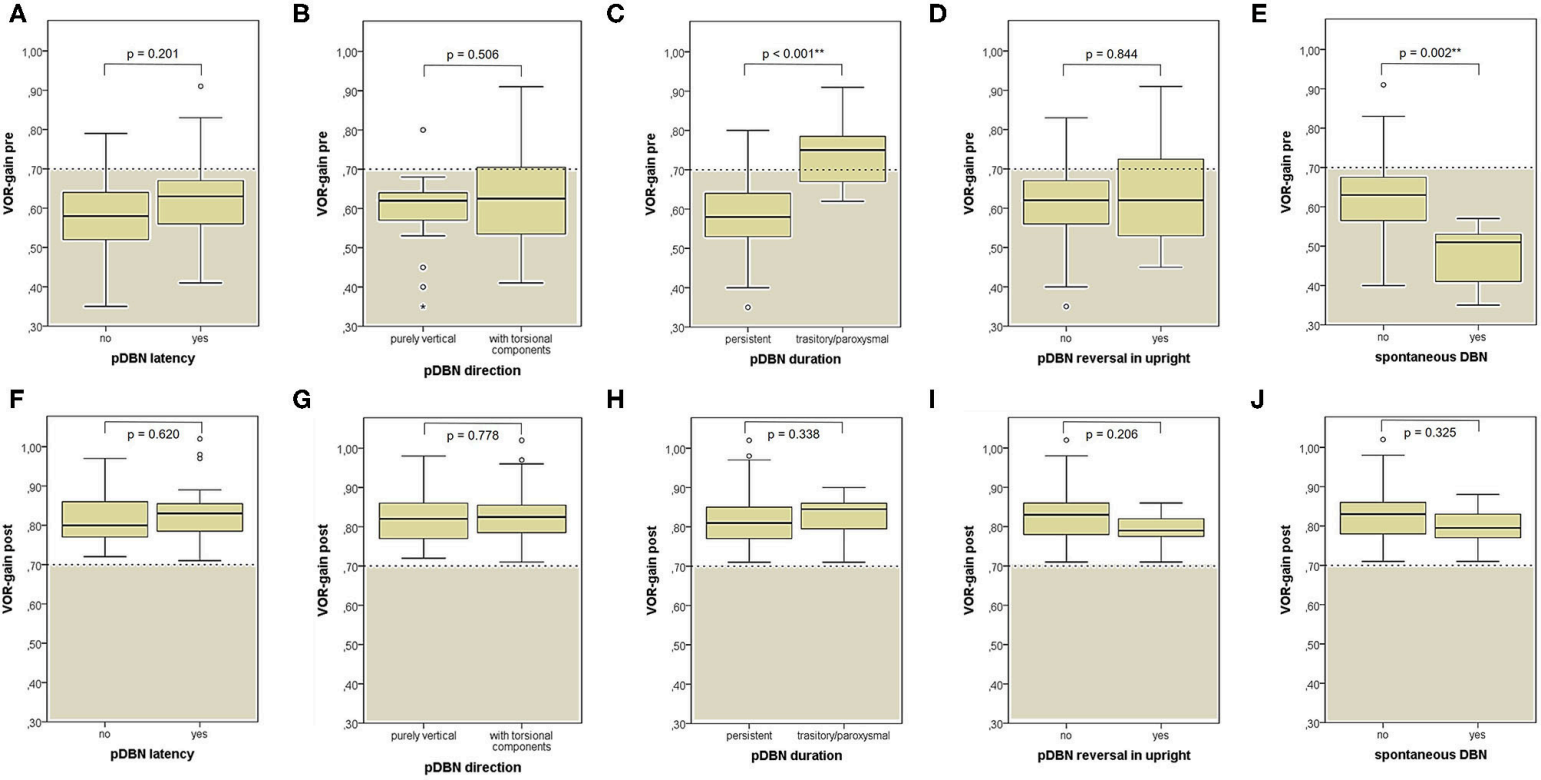
Box plots correlating medians of VOR-gain values at presentation **(A–E)** and following physical treatment **(F–J)** for the SC involved by BPPV among overall subjects (53) with identified affected canal divided based on different VOG findings at presentation. Statistically significant differences at the Mann-Whitney *U*-test are reported and highlighted with **p < 0.01. BPPV, benign paroxysmal positional vertigo; post, post-treatment; pre, at presentation; pDBN, positional downbeat nystagmus; SC, semicircular canal; VOG, video-oculography; VOR, vestibulo-ocular reflex. Values at a greater distance from the median than 1.5 times and 3 times the IQR are plotted individually as dots (weak outliers) and asterisks (strong outliers), respectively.

**Figure 10:**
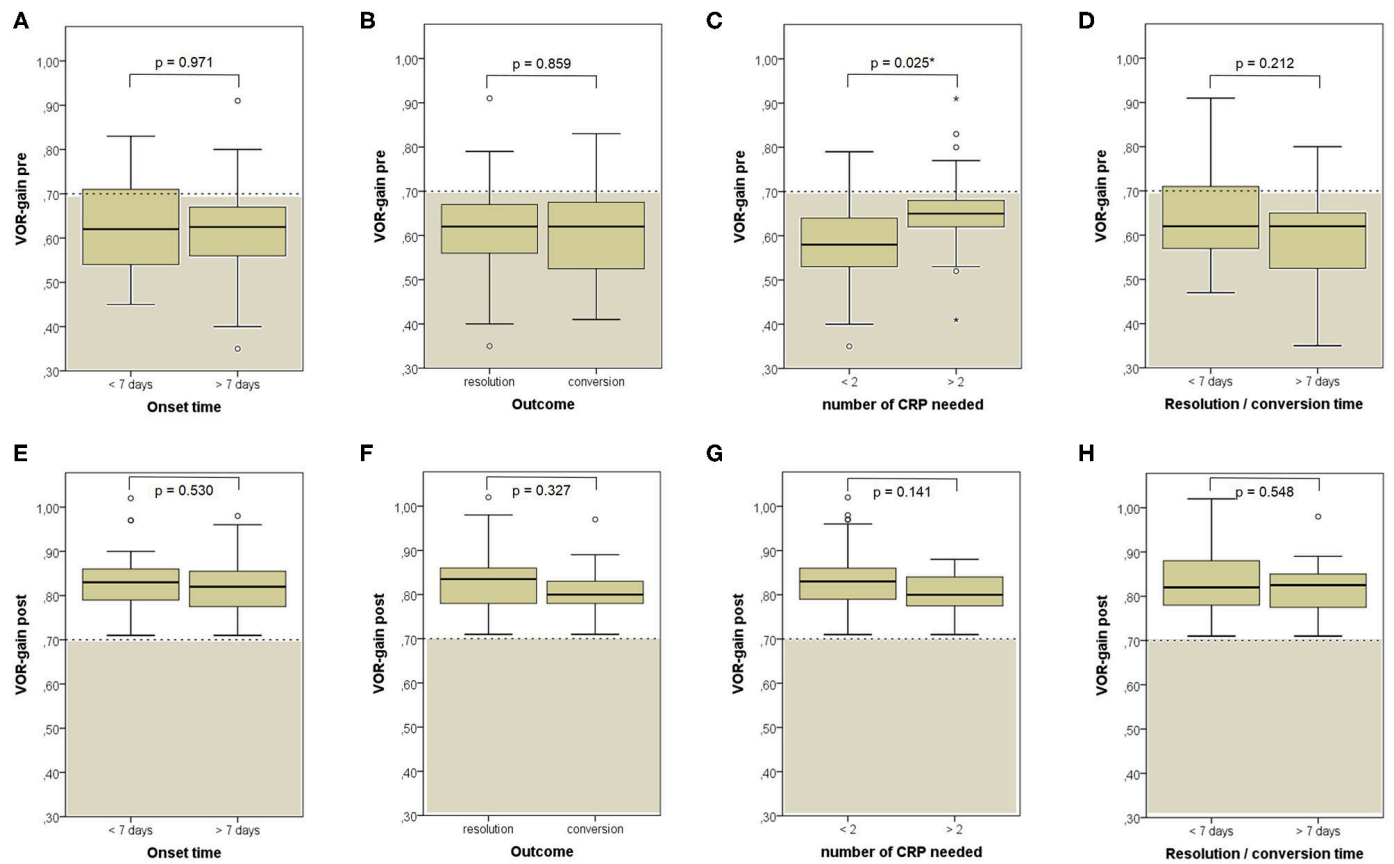
Box plots correlating medians of VOR-gain values at presentation **(A–D)** and following physical treatment **(E–H)** for the SC involved by BPPV among overall subjects (53) with identified affected canal divided based on different details. **(A,E)** Onset time. **(B,F)** Outcome. **(C,G)** Number of CRP needed to restore or convert pDBN. **(D,H)** Time needed for resolution or conversion of pDBN. Statistically significant differences at the Mann-Whitney *U*-test are reported and highlighted with *p < 0.05. BPPV, benign paroxysmal positional vertigo; CRP, canalith repositioning procedure; pDBN, positional downbeat nystagmus; post, post-treatment; pre, at presentation; SC, semicircular canal; VOR, vestibulo-ocular reflex. Values at a greater distance from the median than 1.5 times and 3 times the IQR are plotted individually as dots (weak outliers) and asterisks (strong outliers), respectively.

When exploring variations between presenting and post-treatment VOR-gain values for each SC among overall population with pDBN due to BPPV involving detectable SC (53 cases), a significant functional improvement could be found for the affected SC (*p* < 0.001), for its coupled contralateral canal (p < 0.001) and for the other contralateral vertical SC (*p* = 0.002; Figure 5A). Similar results could be achieved for cases with apogeotropic PSC-BPPV (Figure 5B) and ASC-BPPV (Figure 5C). Increasing of VOR-gain for the affected SC following CRP was more pronounced for cases presenting with persistent pDBN than paroxysmal, irrespective to the type of canal involved (Figure 7A), whereas it was statistically significant only for affected SC presenting with hypoactive canal function (Figure 7B). No significant differences in terms of both presenting and post-treatment values of VOR-gain for the affected SC could be found considering either the type of SC involved (ASC vs. non-ampullary tract of PSC), possible pathologic sides (right vs. left) or different genders (male vs. female). Similar results were achieved dividing overall patients according to previous history of BPPV and head trauma and comparing high-frequency function for the affected SC between subgroups (Figure 8). Conversely, once separated overall population of 53 cases with identified involved canal according to possible pDBN features, presenting VOR-gain values for the affected SC were more severely impaired in cases with persistent positional nystagmus (*p* < 0.001) and with spontaneous DBN (*p* = 0.002) than subjects exhibiting transient/paroxysmal pDBN and lacking of spontaneous nystagmus, respectively (Figures 9A–E). Unlike, no significant disparities in post-treatment VOR-gain values could be found between subgroups with different pDBN characteristics (Figures 9F–J). Functional SC impairment at presentation was also slightly greater in patients successfully treated with one or two CRP than cases requiring more than 2 types of maneuvers either to recover or to convert pDBN into a typical BPPV variant (*p* = 0.025; Figure 10C). On the contrary, presenting VOR-gain for involved canal did not significantly differ considering possible onset times (<7 vs. >7 days), outcomes (resolution vs. conversion) and days needed to treat pDBN (<7 vs. >7 days) (Figures 10A–D), and the same was found comparing VOR-gain values following therapeutic maneuvers among subgroups (Figures 10E–H).

**Figure 11:**
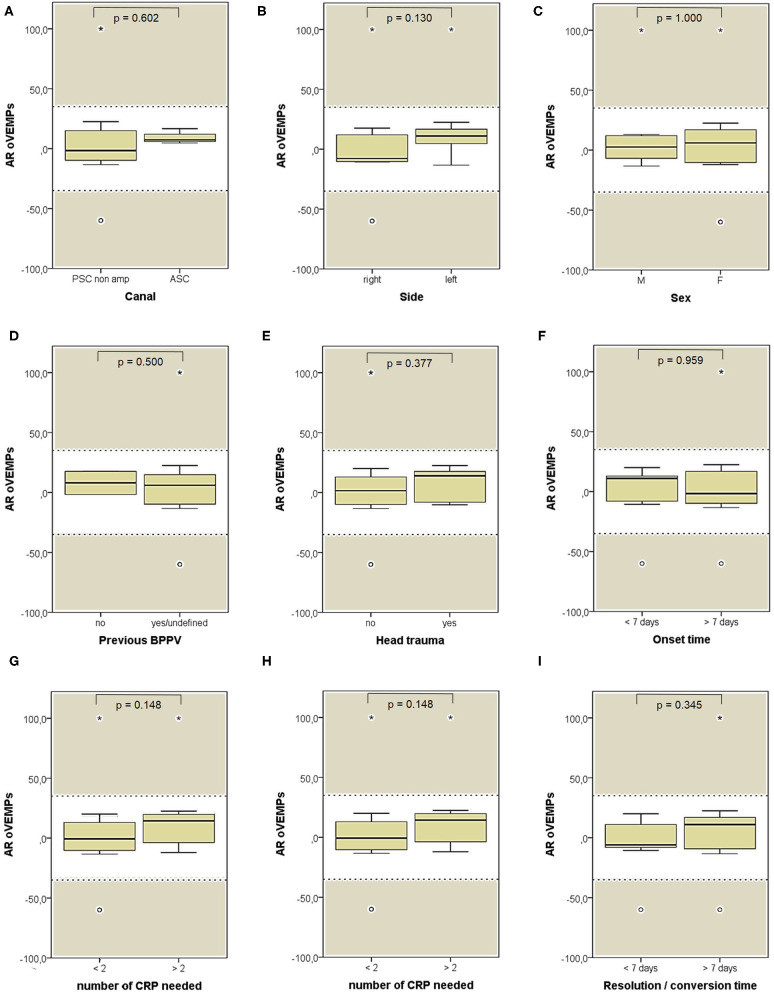
Box plots correlating medians of AR values for air-conducted ocular VEMPs values among 26 patients submitted to electrophysiological testing, divided according to different features. **(A)** Affected semicircular canal. **(B)** Affected side. **(C)** Gender. **(D)** History of recent BPPV. **(E)** History of recent head trauma. **(F)** Onset time. **(G)** Outcome. **(H)** Number of CRP needed to restore or convert pDBN. **(I)** Time needed for resolution or conversion of pDBN. No statistically significant differences at the Mann-Whitney *U*-test are reported. Horizontal dashed lines represent the border between normal and pathologic AR values for ocular VEMPs (35%) and values within gray areas represent abnormal measurements. AR, asymmetry ratio; ASC, anterior semicircular canal; BPPV, benign paroxysmal positional vertigo; CRP, canal repositioning procedures; F, female; M, male; oVEMPs, ocular vestibular-evoked myogenic potentials; pDBN, positional downbeat nystagmus; PSC non-amp, posterior semicircular canal non-ampullary arm. Values at a greater distance from the median than 1.5 times and 3 times the IQR are plotted individually as dots (weak outliers) and asterisks (strong outliers), respectively.

**Figure 12:**
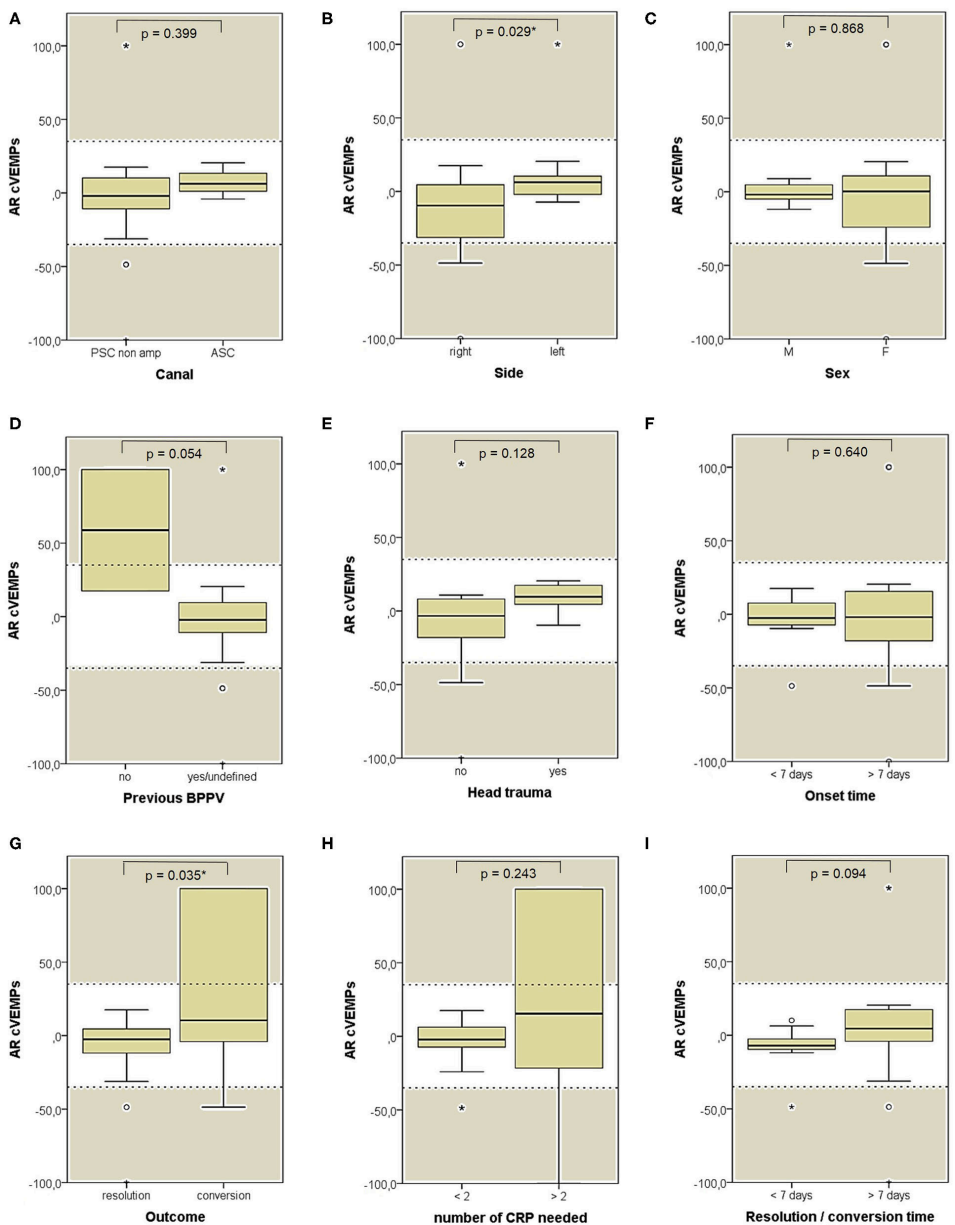
Box plots correlating medians of AR values for air-conducted cervical VEMPs values among 26 patients submitted to electrophysiological testing, divided according to different features. **(A)** Affected semicircular canal. **(B)** Affected side. **(C)** Gender. **(D)** History of recent BPPV. **(E)** History of recent head trauma. **(F)** Onset time. **(G)** Outcome. **(H)** Number of CRP needed to restore or convert pDBN. **(I)** Time needed for resolution or conversion of pDBN. Statistically significant differences at the Mann-Whitney *U*-test are reported and highlighted with for *p* < 0.05. Horizontal dashed lines represent the border between normal and pathologic AR values for cervical VEMPs (35%) and values within gray areas represent abnormal measurements. AR, asymmetry ratio; ASC, anterior semicircular canal; BPPV, benign paroxysmal positional vertigo; CRP, canal repositioning procedures; cVEMPs, cervical vestibular-evoked myogenic potentials; F, female; M, male; pDBN, positional downbeat nystagmus; PSC non-amp, posterior semicircular canal non-ampullary arm. Statistically significant differences at the Mann-Whitney U-test are reported and highlighted with * for *p* < 0.05. Values at a greater distance from the median than 1.5 times and 3 times the IQR are plotted individually as dots (weak outliers) and asterisks (strong outliers), respectively.

Both cVEMPs and oVEMPs to air-conducted sounds were performed only in 26/59 patients (44%) to test saccular and utricular function, respectively. Whereas, no significant difference in utricular function could be found among subgroups (Figure 11), cases with left-sided BPPV and exhibiting pDBN conversion in paroxysmal nystagmus showed greater cVEMPs AR than cases with BPPV involving the right ears (*p* = 0.029) and with pDBN resolution after CRP (*p* = 0.035), respectively (Figure 12).

## Discussion

BPPV is considered the most frequent disorder among peripheral vestibular pathologies with a high prevalence in adult population. Otoliths detachment from utricular macula is the underlying physiopathological mechanism currently accepted (1). Perturbations in SC dynamics due to dislodges particles gravitating within membranous labyrinth mostly result in rotatory vertigo spells triggered by head position changes. Despite positional short-lasting vertigo represent the distinguishing symptom, BPPV-related signs and symptoms may differ among individuals, mainly depending on the portion of the labyrinth involved and on how dislodged otoconia are disposed, resulting in sometimes challenging clinical scenario. In fact, despite PSC represents the most frequently involved site due to its anatomically inferior location in both supine and upright positions, HSC-BPPV accounts for a considerable rate of patents ranging from 10 to 20% of overall cases (1, 51). Conversely, due to its anti-gravity position, ASC ampullary receptor has been found to be rarely activated by endolymphatic perturbations due to detached otoconia, mainly accounting for <5–10% of cases (1, 11, 12, 15, 51, 52). Moreover, despite it has been demonstrated by microscopic investigations how otoliths may either float within membranous ducts (*canalolithiasis*) or adhere to the cupula (*cupulolithiasis*) (53, 54), it has been also hypothesized that a consistent amount of otoconial fragments may sometimes aggregate in clots remaining entrapped within membranous ducts (*canalith jam*) (55, 56). Since several CRP have been described to treat each possible BPPV variant, precise localization of otoconia within the labyrinth is of pivotal importance for treatment outcome. As vestibular testing have a limited role in BPPV diagnosis, not being even recommended in current clinical practice guidelines (57, 58), otoconia siting has been predominantly relied on combining the above-mentioned notions with both the spatial orientation of the assumed SC involved and principles of gravitational fluid mechanics leading to nystagmus recorded during examination and throughout treatment.

The distinguishing feature of the typical variant of PSC-canalolithiasis (involving its ampullary arm) is represented by paroxysmal upbeat nystagmus with torsional components beating toward the undermost ear, referring to the upper corneal poles, evoked by ipsilateral DH maneuver. Geotropic upbeat nystagmus is disconjugated, with a weaker downward and stronger intorsional slow component in the eye of the pathologic side and a stronger downward and weaker extorsional slow component in the opposite eye, resulting from the transitory activation of ipsilateral PSC-ampulla, since debris within the ampullary arm move away from the cupula during diagnostic maneuvers (59). Resulting ampullofugal endolymphatic flows represent an excitatory stimulus for PSC according to Ewald's laws, explaining the aligning plane and the direction of resulting positional nystagmus. It usually exhibits both a typical crescendo-decrescendo course and limited duration as it recedes once debris have reached the undermost position. Moreover, it shows direction reversal with analogous time characteristics once returning to the upright position due to reflux of otoconia toward PSC ampulla. In accordance with Ewald's laws, the latter nystagmus shows lower amplitude than the former, as it results from ampullopetal flows inhibiting PSC afferents (1, 2). Canalolithiasis involving HSC lead to oculomotor patterns exhibiting similar time features to PSC, though it is mainly elicited by head movements along the yaw plane in the supine position (1, 2). Whereas, HSC-cupulolithiasis has been widely investigated, resulting in persistent direction-changing positional nystagmus aligning with the horizontal plane due to a continuous deflection of the overloaded cupula in lateral positionings, cupulolithiasis involving PSC has been rarely described (60–62). It has been related to persistent positional nystagmus elicited in recumbent positionings with either downbeat or upbeat direction depending on anatomy and head-bending angle, similarly to migrainous subjects with supposed modified density ratio between PSC-cupula and surrounding endolymph (63).

**Figure 13:**
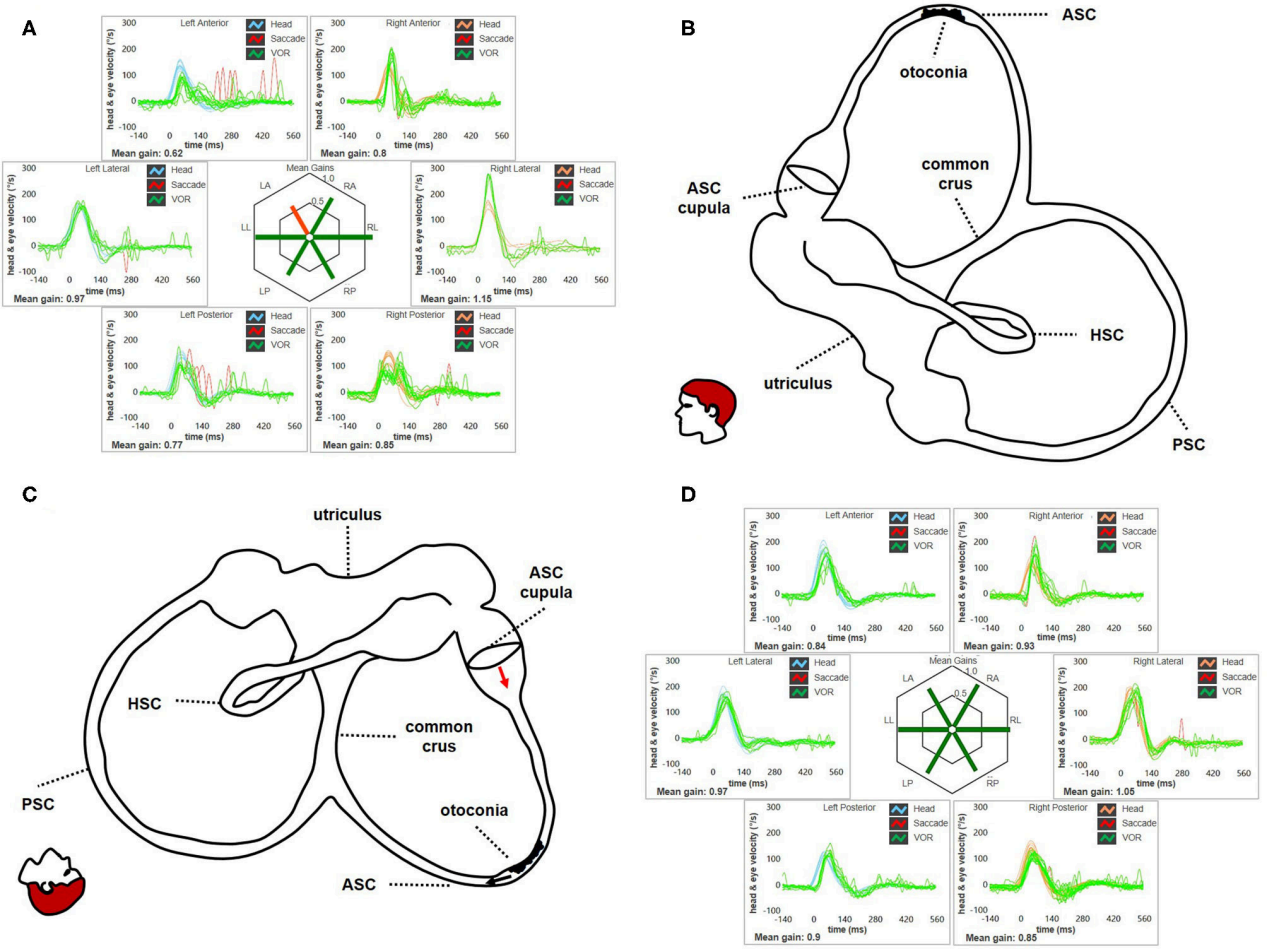
**(A)** Presenting vHIT of subject # 45 affected by BPPV involving the ampullary arm of left ASC. Mean value of VOR-gain (eye velocity/head velocity) is reported for each canal. The hexagonal plot in the center of the figure summarizes mean VOR-gains for each canal; normal gains are shown in green and deficient gains are in red. Selective slight deficient VOR-gain for left ASC (0.62) with scattered overt saccades can be observed. **(B)** Representation for left membranous labyrinth and patient's head along the sagittal (pitch) plane in upright position to explain the site settled by otoconia at presentation. Debris are located within a nearly horizontal tract of the canal, where possible narrowing or irregularities could allow them to settle without resulting in nystagmus in upright position. In this condition, otoliths are thought to partly occlude the canal lumen resulting in an incomplete canalith jam acting as “low-pass filter” for ampullary receptor, thus preventing high-frequency stimuli as detect by vHIT. **(C)** Representation of the disposition of otoconia after SHH test. In this position, otoconia may slowly shift toward the non-ampullary tract of the canal below (black arrow) due to gravity. Resulting excitatory endolymphatic movement should lead ASC cupula to bend ampullofugally (red arrow), accounting for pDBN in recumbent positions. **(D)** vHIT findings after performing CRP for ASC-BPPV leading to return of particles within the utriculus and resolution of symptoms and signs. VOR-gain value for left ASC is back within normal range (0.84). ASC, anterior semicircular canal; BPPV, benign paroxysmal positional vertigo; CRP, canalith repositioning procedure; HSC, horizontal semicircular canal; LA, left anterior; LL, left lateral; LP, left posterior. pDBN, positional downbeat nystagmus; PSC, posterior semicircular canal; RA, right anterior; RL, right lateral; RP, right posterior; SHH, straight head hanging; vHIT, video-head impulse test; VOR, vestibulo-ocular reflex.

**Figure 14:**
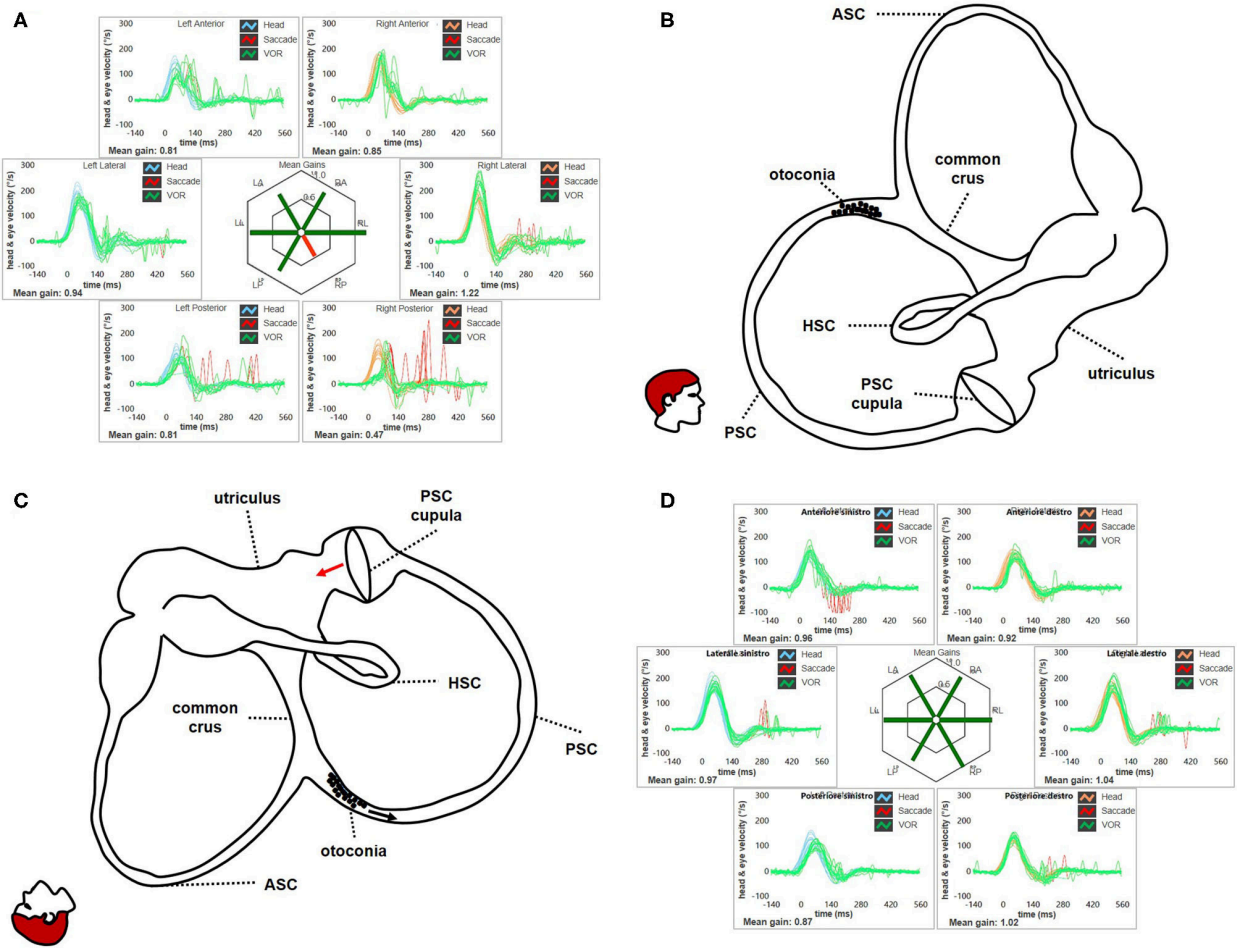
**(A)** Presenting vHIT data for subject # 18 affected by right apogeotropic PSC-BPPV showing selectively impaired VOR-gain values for right PSC (0.47) with both covert and overt saccades. **(B)** Even in this case, otoliths are partially entrapped within a narrow tract of the non-ampullary branch of the canal nearly aligning with the horizontal axis, explaining the lack of spontaneous nystagmus. Likewise ASC, even in this condition the canal is thought to be partially plugged by otoconia, explaining high-frequency VOR-gain impairment for the involved canal. **(C)** Once diagnostic head hanging position is obtained, gravity vector should slowly move debris toward the ampullary tract of the canal below, leading to an ampullopetal endolymphatic flow (black arrow). Resulting cupular bending (red arrow) should generate an inhibitory discharge for PSC afferents, accounting for pDBN. **(D)** vHIT measurement following CRP for apogeotropic PSC-BPPV confirming complete restoration for the impaired PSC VOR-gain. BPPV, benign paroxysmal positional vertigo; CRP, canalith repositioning procedure; HSC, horizontal semicircular canal; LA, left anterior; LL, left lateral; LP, left posterior; pDBN, positional downbeat nystagmus; PSC, posterior semicircular canal; RA, right anterior; RL, right lateral; RP, right posterior; vHIT, video-head impulse test; VOR, vestibulo-ocular reflex.

Nevertheless, most authors have advocated BPPV involving ASC ampullary arm as underlying mechanism for pDBN (Figures 13A–D). In this condition, debris are thought to move away from ASC ampulla resulting in ampullofugal cupular deflection with an excitatory discharge of the superior ampullary nerve [(1, 9, 28, 52); Figure 13C]. Morphological characteristics of pDBN resulting from such a physiological event should exhibit a fast phase torsional components directed toward the affected ear as nystagmus is generated by the contraction of ipsilateral superior rectus and contralateral inferior oblique muscles. Nevertheless, interpretation of pDBN still represent a challenging topic, as patients with ASC-BPPV usually present with atypical positional nystagmus mimicking central pDBN (1–3, 9). In fact, it is rarely evoked only by ipsilateral positioning and it usually exhibits longer time constant compared to typical BPPV-like nystagmus, lacking of both crescendo-decrescendo course and torsional components (3, 9–15). Moreover, it has been recently hypothesized that the same pDBN could also be generated by particles gravitating through the distal portion of the non-ampullary tract of PSC, close to the common crus [(16–27); Figures 14A–D]. In this condition, provoking maneuvers should move debris toward PSC-ampulla leading to an inhibitory discharge of PSC-afferents, which in turn results in pDBN with torsional components beating toward the contralateral ear (Figure 14C). Likewise ASC-BPPV (9), even in this case the non-ampullary arm of each PSC aligns with gravity enough to move debris in ampullopetal direction in both DH bilaterally and SHH positionings (20). Therefore, the same positional nystagmus resulting from activation of ASC-afferents could be also generated inhibiting contralateral posterior ampullary nerve, which drive contractions of the same ocular muscles (18, 28). Though comparison of amplitude between nystagmus evoked in recumbent positionings and reversed nystagmus once returned upright should theoretically distinguish the two forms, as in each case nystagmus could result either from the excitation (stronger nystagmus) of a SC or inhibition (weaker nystagmus) of contralateral canal and viceversa, in both BPPV variants pDBN do not usually reverse in sitting position. This atypical aspect may be due to the reduced movement of the clot in a restricted tract of the SC laying in an almost horizontal plane with the patient upright [(18); Figures 13B, 14B]. Additionally, pDBN often lacks of torsional components in both ASC-BPPV and apogeotropic PSC-BPPV. Authors advocated several explanation for this finding (8, 9, 11, 19, 20, 64). Basically, they stated that since in the human skull, on average, ASC is closer to the sagittal plane (on average only 41°) than PSC (56°) (65, 66), a much smaller torsional component is expected from ASC stimulation. Additionally, calculations of angular eye velocity vectors derived from known canal geometry show the existence of an upwards bias in vertical slow phase eye velocity (67) Thus, more downbeat than torsional nystagmus is expected from ASC-BPPV (9). The same geometrical and neurophysiological considerations have been considered for pDBN resulting from apogeotropic PSC-BPPV, as PSC likely rotates its axis proceeding from the ampullary to non-ampullary arm, so that the latter becomes closer to the sagittal plane (20). Moreover, as the torsional gain of the human VOR is less than unity, about 0.75 and 0.28–0.5 in response to high- and low-frequency roll head rotations, respectively, torsional components of VOR responses should be smaller than the horizontal and vertical components (64). Nevertheless, even though peripheral pDBN is unanimously accepted to align with a more vertical than torsional plane and not to reverse while upright in the majority of cases, these aspects need to be better clarified yet.

According to our original series of 93 patients, most pDBN recognize a peripheral origin confirming previous studies (8, 9, 15). Despite confirming that ASC-canalolithiasis represents a rare entity, it is actually possible to occur. Nevertheless, unlike other reports (19), its prevalence is much smaller than apogeotropic PSC-BPPV, accounting for <12% of pDBN due to BPPV compared to 78% for apogeotropic PSC-BPPV (Table 1). Unlike previously reported series (19), ratio of pDBN with latency, with purely vertical direction and with a persistent course did not significantly differ among subgroups, resulting in challenging differential diagnosis when relying only in the interpretation of pDBN characteristics (Figure 2). It is noteworthy that in our series otoconia entrapped within the non-ampullary branch of PSC were more likely to result in persistent positional nystagmus than ASC (Figure 2C). This may be due to the fact that in general population the tract of the posterior canal approaching the common crus could be more frequently narrow than ASC ampullary arm. According to our result, pDBN could be always evoked in bilateral DH and SHH tests in case of ASC involvement compared to PSC where positional nystagmus was detectable only in one DH positioning in 21.7% of cases (Figure 1B).

Despite specific rehabilitative treatments for these BPPV-variants have been designed reporting good results (12, 14, 19, 20, 24, 28, 45, 46, 68, 69), uncertainty regarding the involved SC represents a dilemma when deciding the best therapeutic approach. Authors have proposed to use the efficacy of appropriate physical therapy or the conversion into a classical ipsilateral canalolithiasis to identify the involved canal (18–20, 24). Others have advocated the use of the so-called “pendular maneuver” aiming to shift otoconia toward PSC ampullary arm to detect the affected canal and proceed later with proper repositioning (70).

The importance of a precise detection of the affected SC is reflected in higher number of CRP needed to restore patients with undefined affected SC compared to cases with identified pathologic canal in our series (Figure 1D), with obvious prognostic sequel and related patient's discomfort. Conversely, irrespective to the canal affected, number of CRP needed, outcome and time required for resolution or conversion did not differ among ASC- and apogeotropic PSC-BPPV (Figure 1). High prevalence of efficacy in two-step maneuvers could be explained assuming that partially entrapped otoliths might fragment during the first maneuver and then return back to the utricle following prolonged positionings.

Although objective measures of canal function would be of extreme help in such cases, diagnostic usefulness of vestibular tests in BPPV remains controversial. Previous investigations assessing the feasibility of VEMPs (71–73) and other tests measuring ampullary activity in different frequency domains (74–77) to detect the ear or the canal involved in patients with typical PSC-canalolithiasis have not achieved univocal consensus. Recently, vHIT has been used to assess high-frequency SCs function in a subsample of patient presenting with persistent pDBN due to the vertical SC-BPPV. Whereas VOR-gain proved to be reduced for the involved canal at presentation, it normalized following proper CRP aimed to release the affected canal from otoconia or to transform pDBN in typical paroxysmal upbeat nystagmus (37). Authors have hypothesized that, unlike typical canalolithiasis involving PSC ampullary tract, where particles are free to float along the membranous duct with minimal effect on cupular dynamics during high-frequency testing (75, 77), in cases with ASC-BPPV and apogeotropic PSC-BPPV presenting with persistent pDBN debris could alter endolymphatic dynamics and cupular response mechanisms, resulting in high-frequency VOR deficit for the involved canal. This condition is thought to occur whenever otoconia settle in physiological narrow portions of the canal lumen [such as the distal portion of the non-ampullary branch of PSC, close to the common crus (20)] or in particular sites of altered canal anatomy due to possible structural changes in SCs orientation (9, 13, 46) or to acquired stenosis of membranous ducts [as demonstrated for ASC ampullary arm (10, 78)] or even to irregularities in membranous walls. Given that hydrodynamic models of fluid-filled SC have demonstrated how a pressure amplification occurs as otoconia enter a narrow section of the canal (79, 80), in particular situations it could likely result in an *incomplete canalith jam* (18, 20) leading to impaired ampullary responses for high-frequency range (37). Namely, this condition would behave as a “*low-pass filter”* allowing the cupula to be activated by low-frequency stimuli (otoconial shifts producing pDBN) while impeding the ampullary receptor to respond to high-frequency inputs (head impulses leading to impaired vHIT data). Partial embedment of debris within narrower portion of membranous duct may also account for the usual persistent course of pDBN with smaller frequency compared to typical PSC-canalolithiasis. In fact, hypothesizing that otoconia could remain incompletely entrapped in these canal tracts, a small amount of endolymphatic reflux is expected and fluid column may continue to press against ampullary receptor, resulting in a slower return of the ampullary crest to the resting position than typical BPPV (20). Finally, considering that some patients with refractory BPPV submitted to surgical plug have recently been found to have, on microscopic examination, fragments of otolith membrane and otoconia encased in their gelatinous matrix rather than simply free-floating otoconia (54), it is not hard to assume how these large materials could be trapped in various locations inside membranous SC dampening endolymphatic flows.

These hypothetical conditions significantly diverge from presenting scenario in typical PSC-canalolithiasis, where dislodged debris prove to be freely moving within the canal by the transitory paroxysmal nystagmus with crescendo-decrescendo course evoked in both recumbent positionings and in return upright. In fact, according to investigations on SCs model, when debris enters the membranous canal from ampulla, a transcapular pressure is generated resulting in cupular displacement and nystagmus onset. Once debris settle on canal walls, they have no more effects on the ampullary receptor, unless the clot fills the portion of the canal with a consequent greater effect on cupular dynamics (80). Whereas, the former mechanism may account for the lack of persistent dynamic perturbation of canal activity by dislodged otoconia in classical PSC-canalolithiasis, with consequent missing abnormalities in vestibular tests assessing canal function (75, 77), the latter finding could likely explain the transient VOR-gain impairment for the affected SC in case of pDBN (37).

In our opinion, this hypothetical mechanism represents the most likely explanation for our findings. In fact, abnormal VOR-gain values were detected in 43/59 cases with pDBN due to vertical SC-BPPV, with a sensitivity of vHIT in detecting the affected SC of 72.9%, irrespective to the canal involved (Figure 4A). In all 43 cases, SC presenting with deficient VOR-gain values matched with the canal involved by BPPV, except for subject #12 who was affected by ASC-canalolithiasis despite presenting with ipsilateral impaired PSC VOR-gain. In this case, transitory pDBN with left-torsional components was related to left ASC-BPPV rather than contralateral apogeotropic PSC-BPPV as the patient was treated few days before for a typical variant of left PSC-canalolithiasis. Moreover, she developed deficient VOR-gain value for left PSC, normalizing after proper CRP for ASC-BPPV, suggesting that otoliths, though eliciting superior ampullary afferents, could have dampened dynamic responses for ipsilateral PSC. We hypothesized a common crus-canalolithiasis for this patient, where geometrical abnormalities in canal disposition, such as ASC with large-sized diameter prevailing over PSC in common crus constitution, could occur. Nevertheless, 25.4% of overall patients presented with transitory nystagmus (Figure 2C), suggesting that otoconia may also be freely moving within either ASC or PSC non-ampullary lumen in some cases. Among them, only 26.7% of cases presented with VOR-gain value <0.7 (Figure 6A), confirming how pDBN with longer time constant is mostly related to the “incomplete jam” theory. The two different mechanisms theorized (*canalolithiasis* vs. *incomplete jam*) could likely account for different behavior of pDBN and affected SC activity in these BPPV variants, explaining also how vertical SCs function could have been normal in a series of patient diagnosed with AC-BPPV presenting with mainly transitory pDBN (81). In fact, examining only data of 44 patients presenting with persistent pDBN, where an incomplete jam is thought to happen, diagnostic sensitivity of vHIT increased up to 88.6% (39/44 cases) (Figure 6A). Additionally, when analyzing pDBN features, VOR-gain values for affected SC presenting with persistent pDBN were found significantly more impaired than cases with transitory/paroxysmal pDBN, further confirming different behavior for high-frequency ampullary responses depending on the degree of otoconial entrapment (Figure 9C). Moreover, in all cases (43/59) presenting with impaired VOR-gain for the affected SC, canal function normalized after proper CRP with either resolution or conversion into a typical BPPV, irrespectively to the underlying diagnosis (Figure 5), confirming a strong linkage between abnormalities for high-frequency canal activity and dislodged otoconia consistently with the “incomplete plug” theory. These assumptions are also in accordance with the significant improvement for VOR-gain detected only for SC exhibiting deficient VOR-gain at presentation, whereas normally active SC did not significantly modify VOR measurements after repositioning (Figure 7B).

It is noteworthy that medians of overall VOR-gain values highly significantly improve not only for the affected SC, but also for the contralateral vertical canal functionally coupled with the involved SC, despite presenting with VOR-gain within normal ranges (Figure 5). This is in line with studies on contralesional canal activity following acute vestibular loss showing, on average, slightly reduced VOR-gain also in the healthy side. It is still matter of debate whether only peripheral phenomena may account for this finding (mainly a functional loss of the “push-pull” mechanism) or if also central compensation (mainly cerebellar “shut-down”) could reduce canal activity on the healthy side (82–84). Probably, the latter phenomenon may likely explain reduced VOR-gain values detected also in the other vertical SC contralaterally to the lesion side and in those patients with long-lasting symptoms (the majority in our cohort). Additionally, canal function, despite normal, resulted to slightly improve even for ipsilateral SCs among cases with involved ASC (Figure 5C). Despite this finding may be a result of casualty, given the small-sized cohort of ASC-BPPV cases, it may be assumed that ASC-BPPV could more likely result in a global labyrinthine perturbation compared to apogeotropic PSC-BPPV.

Nevertheless, no differences were found among presenting VOR-gains for the affected SC according to the canal and side involved, to the patients' gender and to previous history of BPPV or head trauma (Figures 8A–E). Similarly, neither different onset time, outcome nor time needed for resolution or conversion of pDBN in paroxysmal nystagmus were found to impact on presenting function for the involved SC (Figures 10A,B,D). Significantly higher VOR-gain at presentation for the affected SC in patients requiring more than 2 CRP to recover or convert into a typical BPPV compared to those treated with a two-steps maneuver could be explained with the fact that cases in the former group were more likely to have normal VOR-gain, resulting in a more difficult localization of otoconia with vHIT (Figure 10C). On the other hand, the routine use of high-frequency measurement of canal function to detect the canal involved may account for the smaller amount of cases with canal switch in our cohort (Figures 1C, 6D) compared to other series where conversion into a typical BPPV variant was used as a diagnostic tool (19, 20, 24, 70). Interestingly, no significant differences in terms of VOR-gain values following CRP could be found among patients exhibiting different time of symptoms onset, outcomes, number of physical treatments and time required for pDBN resolution or conversion (Figures 10E–H), proving how possible residual dizziness in these patients may be ascribed to other than peripheral causes (85).

As previously mentioned, persistent positional vertical/torsional nystagmus (either upbeat or downbeat, depending on anatomy) evoked in DH or SHH positions has been related to PSC-cupulolithiasis (60–62) or to modified density ratio between the PSC-cupula and surrounding endolymph (63). Therefore, it might be reasonable to assume that presenting findings in some patient from our cohort could be ascribed to such mechanism. Nevertheless, we found some discordant issues between VOG/vHIT findings and cupulolithiasis/buoyancy theory that made us leaning toward BPPV involving non-ampullary arm of PSC resulting in an incomplete jam. Firstly, we did not detect any direction-changing nystagmus by modifying head-bending angle in upright position or in contralateral DH positioning. We could only record slight transient nystagmus reversal when returning upright from head hanging positionings in a small subset of patients of our cohort. In a hypothetical case of PSC-cupulolithiasis, we would have expected to find the above-mentioned findings if PSC-cupula had been overloaded by attached otoliths and bent downward (either toward the canal or toward the ampulla, depending on anatomy, and head-bending angle), persistently exciting or inhibiting, respectively, PSC afferents (60, 61). Moreover, unlike what observed, we would also expected to record a neutral head position where the axis of the affected cupula is supposed to align with gravity, suppressing positional nystagmus (60, 63). Additionally, most cases presenting with hypoactive PSC recovered following CRP properly designed for BPPV involving PSC non-ampullary arm, without canal conversion. Conversely, in case of otoconia attached on the side of cupula overlooking the long arm of the canal, whichever maneuver should always be expected to convert pDBN in paroxysmal upbeating nystagmus as debris should necessarily detach and become freely floating within the membranous PSC before returning within the utricle. Moreover, according to mathematical model, cupulolithiasis resulted to require much greater amount of particles compared to canalolithiasis (79, 80), so its conversion into a canalolithiasis should result in strong positional nystagmus that could hardly go unnoticed by patients. Alternatively, debris hypothetically either settling on the opposite side of the cupula or shifting within PSC short arm should theoretically result in worse symptoms while upright rather than in evident nystagmus in DH positioning, unlike what recorded in our cohort of patients (62, 86). Finally, those cases presenting with reduced VOR-gain for ASC normalizing after CRP should have necessarily exhibited endolymphatic perturbations altering the activity of the superior ampullary receptor rather than PSC-cupulolithiasis. Although other possible explanations could be assumed, in our opinion all these findings suggested that PSC-cupulolithiasis was less likely to occur than apogeotropic PSC-BPPV.

Finally, when considering different VOR behavior for the affected canal between cases exhibiting spontaneous DBN or not, the former group presented with highly reduced function compared to the latter (Figure 9E). This data are in accordance with the assumed mechanism consistent with a canalith jam, where an otoconial clot is thought to completely plug a narrow portion of the membranous duct, blocking endolymphatic flows (56). In this condition, a continuous alteration of hydrostatic pressure between the otoliths clump and the cupula may occur, leading to a persistent cupular deflection thus explaining sudden conversion of positional nystagmus in stationary nystagmus irrespective to head positions occurring during physical treatment (55, 56). As already described for HSC-canalith jam (87, 88), this mechanism may prevent both high- and low-frequency responses for the SC affected (namely head impulses and otoconial shifts, respectively) by blocking endolymphatic flows, likewise surgical plugging (89), thus explaining severe impairment of canal VOR-gain. Though canalith jam has been described for HSC in several reports, occurring either spontaneously (87, 88, 90, 91) or as a result of inappropriate CRP (92–94), a similar condition involving PSC has been recently implied as the hypothetical pathomechanism for spontaneous DBN receding after proper physical treatment (95). Whereas, spontaneous nystagmus resulting from HSC-canalith jam overlaps presenting signs of acute vestibular loss, spontaneous VOG findings due to a PSC involvement should be mainly torsional/vertical aligning with vertical SC axis, thus mimicking CNS pathologies. In these conditions, instrumental equipment for vestibular testing may play a key role in the differential diagnosis, since other end-organs dysfunctions or additional signs of central origin should always coexist with VOR-gain impairment for vertical SC in CNS disorders (36). In Table 2 each possible scenario (*regular canalolithiasis* vs. *incomplete jam* vs. *complete canalith jam*) accounting for different patterns of pDBN due to BPPV and vHIT measurements with corresponding assumed pathomechanism is summarized.

**Table 2:** Table summarizing each of the three possible scenarios accounting for different patterns of pDBN in BPPV and vHIT measurements with corresponding hypothetical pathomechanisms.

	**Oculomotor findings**	**VOR-gain for the affected SC on vHIT**	**Assumed underlying mechanism**	**Endolymphatic flows**	
				**Low-frequency**	**High-frequency**
Regular canalolithiasis	Transient paroxysmal pDBN in DH or SHH, usually reversing in upright	Usually normal	Debris are free to float along the SC	Preserved as debris can move in both directions along the canal	Preserved as debris neither aggregate nor occlude the canal lumen, thus do not impair cupular responses
“Incomplete” (or “functional” or “positional”) canalith jam	Persistent pDBN in DH or SHH, rarely reversing in upright	Slightly reduced	Otoliths are partly entrapped in a narrower canal tract, partially plugging the affected SC lumen	Likely preserved as otoliths, despite partly blocked, are allowed to slowly move toward the cupula in DH or SHH	Impaired as otoliths likely prevent high acceleration flows dampening head impulse responses (behaving as a “low-pass filter”)
Complete canalith jam	Spontaneous DBN, slightly increasing in DH and SHH	Greatly reduced	An otolith clot is completely entrapped within a narrower tract of the canal, entirely plugging the affected SC lumen	Impeded due to a continuous endolymphatic pressure constantly displacing the cupula of the affected SC	Impeded due to a continuous endolymphatic pressure constantly displacing the cupula of the affected SC

*BPPV, benign paroxysmal positional vertigo; DBN, downbeat nystagmus; DH, Dix Hallpike; pDBN, positional downbeat nystagmus; SC, semicircular canal; SHH, straight head hanging; vHIT, video-head impulse test; VOR, vestibulo-ocular reflex*.

Despite our instrumental assessment included both cervical and ocular-VEMPs to air-conducted sounds, they were neither routinely performed to search for possible AR differences among patients nor they were routinely tested both before and after CRP to look for amplitudes changes following proper repositioning. Moreover, due to the lack of bone-conducted stimuli in our equipment, a reliable measurement of both saccular and utricular function could not be obtained. On the other and, analysis of variations in VEMPs amplitudes among BPPV patients would have gone beyond the aim of our investigations. These methodological bias could likely account for the lack of statistically significant results among VEMPs data (Figures 11, 12).

Our investigation presents some other limitations. First of all, being a multicentre investigation, each involved otoneurologist collected data by him/herself, and an inter-observer agreement for ambiguous cases (in particular for three-dimensional evaluation of nystagmus) was never used. Then, despite corrective saccades were always checked to avoid artifacts inclusion in hypoactive VOR-gain plots, our analysis on vHIT data focused almost solely on SC VOR-gain values, whereas morphological study of saccades (covert vs. overt, latency, distribution, peak velocity and inter-aural differences, etc.) was not pursued, being beyond the aims of this study. Moreover, we considered as deficient only VOR-gains below normative data without considering gain asymmetry between coupled pairs of SC. In addition, the subgroup of patients with ASC involvement was significantly smaller than population with apogeotropic PSC-BPPV, leading to possible misleading conclusions when comparing subsamples data. The inclusion in the analysis of 6 subject where the affected SC could not be identified could also have altered final results of our investigation. Though they presented with pDBN exhibiting the same characteristics of the remainder of cases with BPPV and restored with positionings, it could not be excluded they were not affected by vertical SC-BPPV. Finally, despite our cohort size with pDBN collected in a 1-year period was similar to others series, the small-sized sample analyzed does not permit to achieve definitive conclusions on the sensitivity of vHIT in detecting the affected canal in these patients. Further prospective investigations with a more significant number of subjects with pDBN will be needed to better determine the role of vHIT in vertical canals BPPV presenting with pDBN.

## Conclusions

According to our data, vHIT may play a key role in the diagnosis of the affected canal in BPPV involving vertical SC presenting with pDBN. In fact, conversely to typical BPPV with paroxysmal positional nystagmus, where particles are free to move along the membranous ducts, in case of persistent pDBN an *incomplete jam* is likely to occur. Unlike complete canalith jam, where otoconia are thought to plug the entire canal lumen impeding endolymphatic flows for both high- and low-frequency domains due to a continuous pressure exerted by the clot on a persistently displaced cupula, in these conditions particles partially entrapped within a narrow portion of the canal likely behave as a “*low-pass filter”* for the ampullary receptor. This phenomenon may actually impair high-frequency dynamic responses for the affected canal while allowing low-frequency endolymphatic movements, thus explaining reduced VOR-gain for the affected SC on vHIT despite pDBN, respectively, and normalization of head impulse data following symptoms resolution or pDBN conversion into a typical paroxysmal nystagmus. These findings should encourage clinicians to routinely use the vHIT in case of pDBN, including high-frequency testing of canal function in the test battery for these patients, particularly in cases lacking of torsional components, where detection of the affected canal and differential diagnosis with CNS disorders may be challenging.

## Data Availability Statement

All datasets generated for this study are included in the article/Supplementary Material.

## Ethics Statement

The studies involving human participants were reviewed and approved by Area Vasta Nord Emilia Romagna Institutional Review Committee. The patients/participants provided their written informed consent to participate in this study.

## Author Contributions

AC, PM, and SM led the conception of the study and conducted most data acquisition, interpretation and made significant contributions to the writing, and editing of the manuscript. AC conducted data analysis and creation of figures. CB and MR were involved in project conception and manuscript editing. SD, SQ, ER, and EA contributed to data acquisition and manuscript review. EA, MM, AG, and GL were involved in manuscript review. All authors approved the final version of the manuscript.

## Conflict of Interest

The authors declare that the research was conducted in the absence of any commercial or financial relationships that could be construed as a potential conflict of interest.

## Abbreviations

ASC: anterior semicircular canal
BPPV: benign paroxysmal positional vertigo
CRP: canalith repositioning procedure
CNS: central nervous system
DH: Dix Hallpike
DS: Demi Semont
FPP: forced prolonged position
HSC: horizontal semicircular canal
pDBN: positional downbeat nystagmus
PSC: posterior semicircular canal
SC: semicircular canal
SHH: straight head hanging
VEMPs: vestibular-evoked myogenic potentials
vHIT: video-head impulse test
VOG: video-oculography
VOR: vestibulo-ocular reflex.
