# Supplementary material for: Feasibility of Using the Video-Head Impulse Test to Detect the Involved Canal in Benign Paroxysmal Positional Vertigo Presenting With Positional Downbeat Nystagmus
Source: Front Neurol. 2020 Oct 15;11:578588. doi: 10.3389/fneur.2020.578588 (PMC7593380; doi:10.3389/fneur.2020.578588)
Supplement: Supplementary file 1 [file Data_Sheet_1.docx]

**Supplementary file.** Detailed information about all cases included in the study

| **Table A.** Detailed information about personal data, history and presenting instrumental findings of each case included in the analysis (n=59) | | | | | | | | | | | | | | | | | | | | |
| --- | --- | --- | --- | --- | --- | --- | --- | --- | --- | --- | --- | --- | --- | --- | --- | --- | --- | --- | --- | --- |
| **ID** | **Sex** | **Age**  (y) | **Side** | **Affected SC** | **history** | | | | **presenting scenario** | | | | | | | | | | | |
|  |  |  |  |  | **Onset time** | **Previous BPPV** | | **Previous head trauma** | **VOG findings** | | | | | | **VOR-gain on vHIT** | | | | | |
|  |  |  |  |  |  |  |  |  | **spont DBN direction** | **pDBN latency** | **pDBN direction** | **pDBN duration** | **pDBN reversal in upright** | **Provoking positions** | **affected SC** | **ipsi HSC** | **other ipsi vertical SC** | **contra SC coupled with affected SC** | **contra HSC** | **other contra vertical SC** |
| 1 | F | 55 | L | PSC non amp | < 7 d | Undefined | No | | No | Yes | R | Per | No | R-DH | 0,64 | 0,93 | 1,05 | 0,93 | 1 | 0,89 |
| 2 | F | 58 | R | PSC non amp | < 7 d | No | Yes | | No | Yes | L | Per | Yes | B-DH SHH | 0,5 | 1,09 | 1,04 | 0,9 | 0,93 | 0,84 |
| 3 | F | 43 | L | ASC | > 7 d | L-PSC | No | | No | Yes | V | Per | No | B-DH SHH | 0,57 | 0,84 | 0,77 | 0,81 | 0,93 | 0,91 |
| 4 | F | 41 | R | PSC non amp | < 7 d | R-PSC | No | | R | No | R | Per | No | B-DH SHH | 0,53 | 1,03 | 0,97 | 0,93 | 0,88 | 0,74 |
| 5 | F | 54 | R | PSC non amp | < 7 d | Undefined | No | | No | Yes | V | Per | No | B-DH SHH | 0,62 | 1 | 0,89 | 0,83 | 0,84 | 0,82 |
| 6 | F | 54 | R | PSC non amp | > 7 d | R-LSC R-PSC | No | | V | No | V | Per | No | B-DH SHH | 0,35 | 0,98 | 0,89 | 0,71 | 0,83 | 0,73 |
| 7 | M | 46 | L | PSC non amp | > 7 d | Undefined | No | | No | No | V | Per | No | B-DH SHH | 0,63 | 0,94 | 0,81 | 0,7 | 1,09 | 0,89 |
| 8 | M | 34 | L | PSC non amp | < 7 d | L-PSC | Yes | | No | Yes | R | Per | No | B-DH SHH | 0,54 | 1,04 | 0,87 | 0,95 | 1,15 | 0,7 |
| 9 | F | 44 | R | PSC non amp | > 7 d | Undefined | Yes | | No | Yes | V | Per | No | B-DH SHH | 0,63 | 1,14 | 1,08 | 0,89 | 1,06 | 0,7 |
| 10 | M | 58 | L | PSC non amp | > 7 d | Undefined | No | | No | No | V | Per | No | B-DH SHH | 0,4 | 0,95 | 0,79 | 0,84 | 1,03 | 0,7 |
| 11 | M | 48 | L | PSC non amp | < 7 d | L-PSC | No | | No | Yes | R | Per | No | B-DH SHH | 0,56 | 0,87 | 0,95 | 0,87 | 1,05 | 0,91 |
| 12 | F | 48 | L | ASC | < 7 d | L-PSC | No | | No | No | L | Tra | No | B-DH SHH | 0,79 | 0,81 | 0,61 | 0,9 | 0,94 | 0,78 |
| 13 | M | 59 | L | PSC non amp | < 7 d | L-PSC | No | | No | No | R | Per | No | B-DH SHH | 0,6 | 0,9 | 0,82 | 0,84 | 1,02 | 0,79 |
| 14 | F | 70 | L | PSC non amp | < 7 d | L-PSC | No | | V | No | V | Per | No | B-DH SHH | 0,57 | 0,85 | 0,82 | 0,75 | 0,93 | 0,87 |
| 15 | F | 35 | R | PSC non amp | < 7 d | R-PSC | Yes | | No | No | L | Tra | No | B-DH SHH | 0,78 | 0,9 | 0,84 | 0,82 | 0,96 | 0,81 |
| 16 | M | 43 | R | PSC non amp | < 7 d | R-PSC | No | | No | No | L | Tra | No | B-DH SHH | 0,76 | 0,87 | 0,79 | 0,8 | 0,94 | 0,8 |
| 17 | M | 59 | L | PSC non amp | > 7 d | No | No | | No | No | V | Per | No | B-DH SHH | 0,58 | 0,97 | 0,98 | 0,8 | 1,06 | 0,79 |
| 18 | M | 60 | R | PSC non amp | < 7 d | No | No | | No | Yes | L | Per | No | B-DH SHH | 0,47 | 1,22 | 0,85 | 0,81 | 0,94 | 0,81 |
| 19 | M | 28 | R | PSC non amp | > 7 d | R-PSC | No | | No | No | L | Per | No | B-DH SHH | 0,56 | 1 | 0,96 | 0,87 | 0,89 | 0,78 |
| 20 | F | 73 | R | PSC non amp | > 7 d | R-PSC | No | | No | No | L | Per | No | B-DH SHH | 0,52 | 1,03 | 1,22 | 0,75 | 0,96 | 1,31 |
| 21 | M | 71 | R | PSC non amp | > 7 d | R-PSC L-PSC | No | | No | Yes | L | Per | No | B-DH SHH | 0,58 | 0,99 | 0,86 | 0,8 | 0,97 | 0,8 |
| 22 | F | 63 | L | PSC non amp | < 7 d | R-PSC | No | | No | No | V | Per | No | B-DH SHH | 0,64 | 0,9 | 0,82 | 0,86 | 0,94 | 0,83 |
| 23 | M | 64 | R | PSC non amp | < 7 d | Undefined | No | | No | No | L | Per | Yes | B-DH SHH | 0,62 | 0,87 | 0,83 | 0,75 | 0,82 | 0,71 |
| 24 | F | 25 | L | PSC non amp | > 7 d | No | No | | No | No | L | Per | No | B-DH SHH | 0,7 | 0,92 | 0,88 | 0,83 | 0,96 | 0,9 |
| 25 | M | 42 | R | PSC non amp | < 7 d | No | No | | No | No | V | Per | Yes | B-DH SHH | 0,45 | 0,96 | 0,86 | 0,86 | 0,94 | 0,84 |
| 26 | F | 54 | U | Unidentified | < 7 d | Undefined | No | | No | Yes | L | Tra | No | B-DH SHH | No | No | No | No | No | No |
| 27 | F | 48 | U | Unidentified | < 7 d | No | No | | No | No | L | Per | No | B-DH SHH | No | No | No | No | No | No |
| 28 | F | 33 | U | Unidentified | < 7 d | No | No | | No | No | V | Per | No | B-DH SHH | No | No | No | No | No | No |
| 29 | M | 75 | R | PSC non amp | > 7 d | L-LSC L-PSC R-PSC | Yes | | No | Yes | V | Per | No | B-DH SHH | 0,65 | 0,8 | 0,71 | 0,7 | 0,8 | 0,78 |
| 30 | M | 75 | L | ASC | > 7 d | L-LSC L-PSC R-PSC | Yes | | No | Yes | L | Per | Yes | B-DH SHH | 0,56 | 0,8 | 0,77 | 0,76 | 0,83 | 0,91 |
| 31 | F | 88 | L | PSC non amp | < 7 d | L-PSC | No | | No | Yes | R | Tra | Yes | L-DH | 0,74 | 0,84 | 0,7 | 0,93 | 0,96 | 0,7 |
| 32 | F | 78 | L | PSC non amp | > 7 d | Undefined | No | | No | Yes | R | Per | No | B-DH SHH | 0,67 | 0,93 | 0,91 | 0,75 | 1,02 | 0,78 |
| 33 | F | 76 | R | PSC non amp | > 7 d | R-PSC L-PSC | No | | No | Yes | V | Per | No | B-DH SHH | 0,64 | 0,87 | 0,85 | 0,71 | 0,87 | 0,62 |
| 34 | F | 76 | L | PSC non amp | > 7 d | R-PSC L-PSC | No | | No | Yes | V | Per | No | B-DH SHH | 0,62 | 0,87 | 0,71 | 0,85 | 0,87 | 0,64 |
| 35 | M | 54 | U | Unidentified | > 7 d | Undefined | No | | No | Yes | V | Tra | No | B-DH SHH | No | No | No | No | No | No |
| 36 | M | 43 | U | Unidentified | > 7 d | R-PSC L-PSC | Yes | | No | Yes | V | Per | No | B-DH SHH | No | No | No | No | No | No |
| 37 | F | 46 | R | PSC non amp | < 7 d | R-PSC | No | | No | Yes | L | Tra | Yes | R-DH | 0,71 | 1,1 | 0,88 | 0,71 | 1,04 | 0,74 |
| 38 | F | 38 | R | PSC non amp | < 7 d | No | No | | No | Yes | L | Per | No | B-DH SHH | 0,56 | 1,04 | 1,07 | 0,72 | 0,95 | 0,83 |
| 39 | M | 57 | L | PSC non amp | > 7 d | Undefined | No | | R | Yes | R | Per | No | B-DH SHH | 0,52 | 0,98 | 0,82 | 0,74 | 1,09 | 0,79 |
| 40 | F | 53 | L | ASC | > 7 d | No | No | | No | Yes | V | Tra | No | B-DH SHH | 0,62 | 0,84 | 0,78 | 0,7 | 0,92 | 0,81 |
| 41 | M | 52 | U | Unidentified | > 7 d | Undefined | Yes | | No | Yes | V | Tra | No | B-DH SHH | No | No | No | No | No | No |
| 42 | F | 66 | L | ASC | > 7 d | L-PSC | Yes | | No | Yes | V | Per | No | B-DH SHH | 0,53 | 0,89 | 0,92 | 0,78 | 1,02 | 0,73 |
| 43 | F | 54 | L | PSC non amp | < 7 d | No | No | | No | Yes | R | Tra | No | B-DH SHH | 0,83 | 0,89 | 0,76 | 0,92 | 0,98 | 0,81 |
| 44 | M | 74 | L | PSC non amp | > 7 d | Undefined | No | | No | Yes | R | Tra | No | L-DH | 0,77 | 1,07 | 0,87 | 0,91 | 1,27 | 0,71 |
| 45 | M | 69 | L | ASC | > 7 d | L-PSC | No | | No | Yes | V | Tra | No | B-DH SHH | 0,62 | 0,97 | 0,77 | 0,85 | 1,15 | 0,8 |
| 46 | F | 74 | R | PSC non amp | > 7 d | Undefined | No | | No | Yes | L | Per | No | R-DH | 0,48 | 0,97 | 0,88 | 0,7 | 0,95 | 0,71 |
| 47 | F | 67 | R | PSC non amp | < 7 d | No | No | | L | No | L | Per | No | B-DH SHH | 0,5 | 0,9 | 0,76 | 0,74 | 0,87 | 0,72 |
| 48 | F | 46 | L | PSC non amp | > 7 d | L-LSC L-PSC | Yes | | No | Yes | R | Per | No | B-DH SHH | 0,68 | 0,92 | 0,96 | 0,88 | 1,05 | 0,84 |
| 49 | F | 63 | R | PSC non amp | > 7 d | No | No | | L | Yes | L | Per | No | B-DH SHH | 0,41 | 0,88 | 1 | 0,71 | 0,8 | 0,93 |
| 50 | F | 65 | R | PSC non amp | < 7 d | No | No | | No | Yes | V | Per | No | B-DH SHH | 0,65 | 0,98 | 0,78 | 0,7 | 0,92 | 0,73 |
| 51 | F | 62 | R | PSC non amp | > 7 d | L-PSC R-PSC | No | | No | Yes | V | Per | No | B-DH SHH | 0,68 | 1,05 | 0,87 | 0,78 | 0,81 | 0,8 |
| 52 | F | 64 | L | ASC | > 7 d | L-PSC R-PSC | No | | No | Yes | L | Tra | No | B-DH SHH | 0,67 | 0,87 | 0,87 | 0,78 | 0,96 | 0,82 |
| 53 | M | 51 | L | PSC non amp | > 7 d | R-PSC | No | | No | Yes | R | Tra | No | L-DH | 0,67 | 1,02 | 0,77 | 0,85 | 1,15 | 0,75 |
| 54 | F | 49 | R | PSC non amp | > 7 d | R-PSC | No | | No | Yes | V | Per | No | B-DH SHH | 0,62 | 1 | 0,93 | 0,72 | 0,85 | 0,86 |
| 55 | F | 72 | L | PSC non amp | > 7 d | L-PSC R-PSC | No | | No | Yes | V | Per | No | B-DH SHH | 0,63 | 1,04 | 0,85 | 0,75 | 0,85 | 0,63 |
| 56 | F | 72 | R | PSC non amp | > 7 d | L-PSC R-PSC | No | | No | Yes | L | Per | No | B-DH SHH | 0,63 | 0,85 | 0,75 | 0,85 | 1,04 | 0,63 |
| 57 | F | 70 | R | PSC non amp | > 7 d | R-PSC | No | | No | Yes | L | Per | No | R-DH | 0,65 | 1 | 0,78 | 0,79 | 1,01 | 0,74 |
| 58 | F | 70 | L | PSC non amp | > 7 d | R-PSC | No | | No | Yes | V | Per | No | B-DH SHH | 0,8 | 1,01 | 0,92 | 0,82 | 1,13 | 0,83 |
| 59 | F | 56 | R | PSC non amp | > 7 d | R-PSC | No | | No | Yes | L | Tra | Yes | R-DH | 0,91 | 1,12 | 0,84 | 0,72 | 0,94 | 0,93 |

**Abbreviations.**

**Age:** patient's age

**Sex:** patient’s sex (F: female, M: male)

**Side:** side involved (L: left, R: right, U: unidentified)

**Affected SC:** semicircular canal involved by BPPV (ASC: anterior semicircular canal, PSC non amp: posterior semicircular canal non-ampullary arm, unidentified: unidentified canal)

**Onset time:** days from symptoms onset and presentation (< 7 d: less than 7 days, > 7 d: more than 7 days)

**Previous BPPV:** ascertained episode of BPPV within 30 days prior to presentation (L-ASC: left anterior semicircular canal, L-LSC: left lateral semicircular canal, L-PSC: left posterior semicircular canal, R-ASC: right anterior semicircular canal, R-LSC: right lateral semicircular canal, R-PSC: right posterior semicircular canal, no: no previous BPPV, undefined: undefined previous BPPV)

**Previous head trauma:** recent head trauma (Yes: recent trauma, No: no recent trauma)

**spont DBN direction:** direction of spontaneous downbeating nystagmus (V: purely vertical, L: with left-beating components i.e. with the upper pole of the eyes rotating toward patients’ left ear, No: no spontaneous nystagmus, R: with right-beating components i.e. with the upper pole of the eyes rotating toward patients’ right ear)

**pDBN latency:** latency of positional downbeat nystagmus (Yes: latent, No: not latent)

**pBDN direction:** direction of positional downbeat nystagmus (V: purely vertical, L: with left-beating components i.e. with the upper pole of the eyes rotating toward patients’ left ear, R: with right-beating components i.e. with the upper pole of the eyes rotating toward patients’ right ear)

**pDBN duration:** duration of positional downbeat nystagmus (Per: persistent nystagmus lasting > 2 min, Tra: transient/paroxysmal nystagmus lasting < 2min)

**pDBN reversal in upright:** reversal of direction of positional downbeat nystagmus from positionings to upright position (Yes: inversion, No: no inversion)

**Provoking positioning:** maneuver/s eliciting positional downbeat ny (R-DH: right Dix Hallpike, L-DH: left Dix Hallpike, B-DH: both Dix Hallpike, SHH: straight head hanging)

**VOR-gain for affected SC:** VOR-gain value for the affected vertical canal at the vHIT (No: canal not identified)

**VOR-gain for ipsi HSC:** VOR-gain value for the horizontal semicircular canal ipsilateral to the affected canal at the vHIT (No: canal not identified)

**VOR-gain for other ipsi vertical SC:** VOR-gain value for the other vertical canal ipsilateral to the affected canal at the vHIT (No: canal not identified)

**VOR-gain for contra SC coupled with affected SC:** VOR-gain value for the vertical canal of the unaffected side coupled to the affected canal at the vHIT (No: canal not identified)

**VOR-gain for contra HSC:** VOR-gain value for the horizontal semicircular canal of the unaffected side at the vHIT (No: canal not identified)

**VOR-gain for other contra vertical SC:** VOR-gain value for the other vertical canal of the unaffected side at the vHIT (No: canal not identified

| **Table B.** Detailed information about personal data, treatment and post-treatment findings of each case included in the analysis (n=59) | | | | | | | | | | | | | | | | | | |
| --- | --- | --- | --- | --- | --- | --- | --- | --- | --- | --- | --- | --- | --- | --- | --- | --- | --- | --- |
| **ID** | **Sex** | **Age**  **(y)** | **Side** | **Affected SC** | **treatment** | | | | **post-treatment findings** | | | | | | | | | |
|  |  |  |  |  | **CRP** | **Outcome** | **BPPV conversion** | **Resol / conv time** | **VOR-gain on vHIT** | | | | | | **cVEMPs** | | **oVEMPs** | |
|  |  |  |  |  |  |  |  |  | **affected SC** | **ipsi HSC** | **other ipsi vertical SC** | **contra SC coupled with affected SC** | **contra HSC** | **other contra vertical SC** | **ipsi** (µV) | **contra** (µV) | **ipsi**  (µV) | **contra** (µV) |
| 1 | F | 55 | L | PSC non amp | R-DS + R-E | Conv | L-PSC amp | < 7 d | 0,97 | 0,96 | 0,96 | 0,82 | 1,04 | 0,85 | 117 | 143,4 | 1 | 1,3 |
| 2 | F | 58 | R | PSC non amp | L-DS + L-FPP | Resol | No | > 7 d | 0,86 | 1,14 | 1,01 | 1,07 | 1,03 | 0,87 | 63,5 | 90,5 | 0,7 | 1 |
| 3 | F | 43 | L | ASC | PFPP + Y | Conv | L-PSC amp | > 7 d | 0,89 | 0,95 | 0,89 | 0,84 | 1,11 | 1,03 | 121,6 | 112 | 5,1 | 5,9 |
| 4 | F | 41 | R | PSC non amp | L-DS | Resol | No | < 7 d | 0,77 | 1 | 0,9 | 0,94 | 0,91 | 0,73 | 82,7 | 71,8 | 2,1 | 1,7 |
| 5 | F | 54 | R | PSC non amp | L-DS + L-FPP | Conv | R-PSC amp | < 7 d | 0,8 | 0,96 | 0,96 | 0,8 | 0,83 | 0,86 | 342 | 118 | 8 | 2 |
| 6 | F | 54 | R | PSC non amp | L-DS + L-FPP | Resol | No | > 7 d | 0,88 | 1,03 | 0,88 | 0,82 | 0,93 | 0,79 | 342 | 118 | 8 | 2 |
| 7 | M | 46 | L | PSC non amp | R-DS + R-E | Resol | No | > 7 d | 0,72 | 1 | 0,82 | 0,78 | 1,11 | 0,84 | 128,2 | 123,4 | 1,7 | 1,3 |
| 8 | M | 34 | L | PSC non amp | R-DS + R-E | Resol | No | > 7 d | 0,85 | 0,98 | 0,94 | 0,99 | 1,12 | 0,77 | 104,1 | 124,7 | 0,4 | 0,5 |
| 9 | F | 44 | R | PSC non amp | L-DS | Resol | No | > 7 d | 0,85 | 1,05 | 0,85 | 0,86 | 0,93 | 0,73 | 105 | 114,8 | 1,6 | 1,3 |
| 10 | M | 58 | L | PSC non amp | R-DS + R-E | Resol | No | > 7 d | 0,82 | 0,97 | 1,02 | 1 | 1,07 | 0,86 | 69,8 | 67,1 | 1,8 | 1,6 |
| 11 | M | 48 | L | PSC non amp | R-DS + R-E | Resol | No | > 7 d | 0,83 | 0,91 | 1,04 | 0,93 | 1,01 | 0,93 | 116 | 117 | 1 | 1,3 |
| 12 | F | 48 | L | ASC | PFPP + Y | Resol | No | < 7 d | 0,9 | 0,9 | 0,8 | 0,83 | 0,91 | 0,86 | 70,5 | 80 | 4,1 | 4,5 |
| 13 | M | 59 | L | PSC non amp | R-DS + R-E | Resol | No | < 7 d | 0,78 | 1 | 0,8 | 0,79 | 0,98 | 0,85 | 81 | 70 | 0,8 | 1 |
| 14 | F | 70 | L | PSC non amp | R-DS + R-E | Conv | L-PSC amp | < 7 d | 0,8 | 0,85 | 0,82 | 0,75 | 0,93 | 0,87 | 120 | 110 | 0,8 | 1,2 |
| 15 | F | 35 | R | PSC non amp | L-DS + L-E | Resol | No | < 7 d | 0,85 | 0,94 | 0,82 | 0,8 | 0,9 | 0,79 | 85 | 70,1 | 2 | 1,7 |
| 16 | M | 43 | R | PSC non amp | L-DS + L-E | Resol | No | < 7 d | 0,79 | 0,9 | 0,8 | 0,83 | 0,93 | 0,85 | 68,3 | 65 | 2,1 | 1,8 |
| 17 | M | 59 | L | PSC non amp | R-DS + R-FPP | Resol | No | < 7 d | 0,74 | 0,97 | 0,87 | 0,73 | 1,07 | 0,85 | n.p. | n.p. | n.p. | n.p. |
| 18 | M | 60 | R | PSC non amp | L-DS + L-FPP | Resol | No | < 7 d | 1,02 | 1,04 | 0,92 | 0,96 | 0,97 | 0,87 | n.p. | n.p. | n.p. | n.p. |
| 19 | M | 28 | R | PSC non amp | L-DS + L-FPP | Resol | No | < 7 d | 0,86 | 1,03 | 0,88 | 0,94 | 0,92 | 0,7 | n.p. | n.p. | n.p. | n.p. |
| 20 | F | 73 | R | PSC non amp | L-DS + L-FPP | Resol | No | > 7 d | 0,74 | 1,05 | 0,82 | 0,76 | 0,94 | 0,7 | n.p. | n.p. | n.p. | n.p. |
| 21 | M | 71 | R | PSC non amp | L-DS + L-E + PFPP + Y | Resol | No | < 7 d | 0,82 | 1,09 | 0,9 | 0,93 | 0,82 | 0,88 | 231,3 | 182,2 | 0,9 | 0,8 |
| 22 | F | 63 | L | PSC non amp | R-DS + R-FPP | Resol | No | < 7 d | 0,97 | 0,9 | 0,83 | 0,92 | 0,95 | 0,89 | n.p. | n.p. | n.p. | n.p. |
| 23 | M | 64 | R | PSC non amp | L-DS + L-FPP | Resol | No | < 7 d | 0,79 | 0,88 | 0,82 | 0,81 | 0,86 | 0,75 | n.p. | n.p. | n.p. | n.p. |
| 24 | F | 25 | L | PSC non amp | R-DS + R-FPP | Resol | L-PSC amp | < 7 d | 0,96 | 0,93 | 0,88 | 0,89 | 0,92 | 0,88 | n.p. | n.p. | n.p. | n.p. |
| 25 | M | 42 | R | PSC non amp | R-E + R-FPP | Conv | R-PSC amp | > 7 d | 0,77 | 0,95 | 0,83 | 0,82 | 0,94 | 0,84 | n.p. | n.p. | n.p. | n.p. |
| 26 | F | 54 | U | Unidentified | L-DS + L-FPP + R-DS + R-FPP + Y | Resol | No | > 7 d | No | No | No | No | no | No | n.p. | n.p. | n.p. | n.p. |
| 27 | F | 48 | U | Unidentified | L-DS + L-FPP + R-DS + R-FPP + Y | Resol | No | > 7 d | No | No | No | No | no | No | n.p. | n.p. | n.p. | n.p. |
| 28 | F | 33 | U | Unidentified | L-DS + L-FPP + R-DS + R-FPP + Y | Resol | No | > 7 d | No | No | No | No | no | No | n.p. | n.p. | n.p. | n.p. |
| 29 | M | 75 | R | PSC non amp | L-E + L-FPP + L-S + PFPP + Y | Resol | No | > 7 d | 0,76 | 0,83 | 0,91 | 0,71 | 0,8 | 0,77 | n.p. | n.p. | n.p. | n.p. |
| 30 | M | 75 | L | ASC | PFPP + Y | Resol | No | > 7 d | 0,84 | 0,84 | 0,9 | 0,8 | 0,92 | 1 | n.p. | n.p. | n.p. | n.p. |
| 31 | F | 88 | L | PSC non amp | R-E + R-FPP | Resol | No | > 7 d | 0,8 | 0,8 | 0,72 | 0,91 | 0,88 | 0,72 | n.p. | n.p. | n.p. | n.p. |
| 32 | F | 78 | L | PSC non amp | R-E + R-FPP | Conv | L-LSC non amp | > 7 d | 0,81 | 0,88 | 0,75 | 0,74 | 1 | 0,72 | n.p. | n.p. | n.p. | n.p. |
| 33 | F | 76 | R | PSC non amp | L-E + L-FPP | Resol | No | > 7 d | 0,86 | 0,98 | 0,95 | 0,85 | 0,87 | 0,98 | n.p. | n.p. | n.p. | n.p. |
| 34 | F | 76 | L | PSC non amp | R-E + R-FPP | Resol | No | > 7 d | 0,98 | 0,87 | 0,85 | 0,95 | 0,98 | 0,86 | n.p. | n.p. | n.p. | n.p. |
| 35 | M | 54 | U | Unidentified | L-E + R-E + Y | Resol | No | > 7 d | No | No | No | No | no | No | n.p. | n.p. | n.p. | n.p. |
| 36 | M | 43 | U | Unidentified | L-S + R-S + PFPP + Y | Resol | No | > 7 d | No | No | No | No | no | No | n.p. | n.p. | n.p. | n.p. |
| 37 | F | 46 | R | PSC non amp | L-DS + L-E | Resol | No | < 7 d | 0,71 | 1,17 | 0,86 | 0,79 | 1,06 | 0,8 | n.p. | n.p. | n.p. | n.p. |
| 38 | F | 38 | R | PSC non amp | L-DS + L-FPP | Resol | No | < 7 d | 0,76 | 1,09 | 0,74 | 0,8 | 0,97 | 0,72 | n.p. | n.p. | n.p. | n.p. |
| 39 | M | 57 | L | PSC non amp | R-DS + R-E + R-FPP | Conv | L-LSC amp | > 7 d | 0,71 | 0,94 | 0,78 | 0,93 | 1,09 | 0,74 | 0 | 150 | 0 | 5,8 |
| 40 | F | 53 | L | ASC | L-E + PFPP + Y | Resol | No | > 7 d | 0,86 | 0,86 | 0,86 | 0,79 | 0,98 | 0,89 | n.p. | n.p. | n.p. | n.p. |
| 41 | M | 52 | U | Unidentified | L-E + L-S + R-E + R-S | Resol | No | > 7 d | No | No | No | No | no | No | n.p. | n.p. | n.p. | n.p. |
| 42 | F | 66 | L | ASC | PFPP + Y | Conv | L-LSC amp | > 7 d | 0,76 | 0,96 | 1,02 | 0,75 | 1,17 | 0,96 | 67,5 | 102 | 6,5 | 9,1 |
| 43 | F | 54 | L | PSC non amp | R-DS + R-E + R-FPP + Y | Conv | L-PSC amp | < 7 d | 0,88 | 0,84 | 0,72 | 0,9 | 0,92 | 0,7 | n.p. | n.p. | n.p. | n.p. |
| 44 | M | 74 | L | PSC non amp | R-E + R-FPP + Y | Conv | L-LSC non amp | > 7 d | 0,83 | 1,22 | 0,74 | 0,9 | 1,31 | 0,72 | n.p. | n.p. | n.p. | n.p. |
| 45 | M | 69 | L | ASC | L-E + Y + PFPP | Resol | No | > 7 d | 0,84 | 0,97 | 0,9 | 0,85 | 1,05 | 0,93 | n.p. | n.p. | n.p. | n.p. |
| 46 | F | 74 | R | PSC non amp | L-E + L-FPP | Resol | No | > 7 d | 0,85 | 0,99 | 0,83 | 0,88 | 0,87 | 0,72 | 46,1 | 57,3 | 0 | 1,7 |
| 47 | F | 67 | R | PSC non amp | L-DS + L-FPP | Conv | R-PSC amp | < 7 d | 0,83 | 1 | 0,94 | 0,75 | 0,89 | 0,87 | n.p. | n.p. | n.p. | n.p. |
| 48 | F | 46 | L | PSC non amp | R-DS + R-E + R-FPP + R-S + Y | Conv | L-LSC non amp | > 7 d | 0,8 | 0,88 | 0,87 | 0,87 | 0,99 | 0,8 | 55,1 | 67,9 | 4,3 | 6,8 |
| 49 | F | 63 | R | PSC non amp | L-DS + L-S + L-FPP | Conv | R-PSC amp | > 7 d | 0,79 | 0,9 | 1 | 0,7 | 0,8 | 0,96 | 0 | 32,9 | 3,1 | 3 |
| 50 | F | 65 | R | PSC non amp | L-DS + L-S + L-FPP + Y | Resol | No | > 7 d | 0,84 | 0,97 | 0,83 | 0,86 | 0,91 | 0,78 | n.p. | n.p. | n.p. | n.p. |
| 51 | F | 62 | R | PSC non amp | L-DS + L-E + L-FPP | Resol | No | > 7 d | 0,83 | 1,02 | 0,78 | 0,93 | 0,88 | 0,83 | n.p. | n.p. | n.p. | n.p. |
| 52 | F | 64 | L | ASC | L-S + R-S + Y | Resol | No | < 7 d | 0,85 | 0,89 | 0,85 | 0,81 | 0,96 | 0,83 | n.p. | n.p. | n.p. | n.p. |
| 53 | M | 51 | L | PSC non amp | R-E + R-S + R-FPP | Resol | No | > 7 d | 0,86 | 1,12 | 0,81 | 0,83 | 1,13 | 0,88 | n.p. | n.p. | n.p. | n.p. |
| 54 | F | 49 | R | PSC non amp | L-DS + L-E + L-FPP + L-S | Resol | No | > 7 d | 0,77 | 1,01 | 1 | 0,79 | 0,82 | 0,92 | 161 | 84,4 | 6,6 | 9,3 |
| 55 | F | 72 | L | PSC non amp | L-E + L-FPP + L-S | Conv | L-PSC amp | > 7 d | 0,79 | 0,9 | 0,83 | 0,89 | 0,93 | 0,78 | 0 | 24 | 2,8 | 2,2 |
| 56 | F | 72 | R | PSC non amp | R-E + R-FPP + R-S | Resol | No | > 7 d | 0,78 | 0,93 | 0,89 | 0,83 | 0,9 | 0,79 | 24 | 0 | 2,2 | 2,8 |
| 57 | F | 70 | R | PSC non amp | L-E + L-FPP | Resol | No | > 7 d | 0,8 | 1,13 | 0,76 | 1,01 | 1,03 | 0,74 | 175 | 107 | 7,1 | 5,9 |
| 58 | F | 70 | L | PSC non amp | R-E + R-FPP + Y | Conv | L-PSC amp | > 7 d | 0,76 | 0,94 | 0,86 | 0,82 | 1,1 | 0,81 | n.p. | n.p. | n.p. | n.p. |
| 59 | F | 56 | R | PSC non amp | L-FPP + L-S | Resol | No | < 7 d | 0,78 | 1 | 0,87 | 0,74 | 0,95 | 0,86 | n.p. | n.p. | n.p. | n.p. |

**Abbreviations.**

**Age:** patient's age

**Sex:** patient’s sex (F: female, M: male)

**Side:** side involved (R: right, L: left, U: unidentified)

**Affected SC:** semicircular canal involved by BPPV (ASC: anterior semicircular canal, PSC non amp: posterior semicircular canal non-ampullary arm, unidentified: unidentified canal)

**CRP:** canal repositioning procedure performed (L-DS: leftward Demi-Semont, L-E: leftward Epley, L-FPP: leftside forced prolonged position, L-S: leftward Semont, PFPP: prolonged forced position procedure, R-DS: rightward Demi-Semont, R-E: rightward Epley, R-FPP: rightside forced prolonged position, R-S: rightward Semont, Y: Yacovino)

**Outcome:** outcome of canal repositioning procedure performed (Conv: conversion, Resol: resolution)

**BPPV conversion:** conversion to more typical BPPV involving other canals or other arms (L-ASC: left anterior semicircular canal, L-LSC amp: left lateral semicircular canal ampullary arm, L-LSC non amp: left lateral semicircular canal non-ampullary arm, L-PSC amp: left posterior semicircular canal ampullary arm, R-ASC: right anterior semicircular canal, R-LSC amp: right lateral semicircular canal ampullary arm, R-LSC non amp: right lateral semicircular canal non-ampullary arm, R-PSC amp: right posterior semicircular canal ampullary arm, No: no canal conversion)

**Resol/convers time:** time from beginning of canal repositioning procedures to conversion/resolution (< 7 d: less than 7 days, > 7 d: more than 7 days)

**VOR-gain for affected SC:** VOR-gain value for the affected vertical canal at the vHIT (No: canal not identified)

**VOR-gain for ipsi HSC:** VOR-gain value for the horizontal semicircular canal ipsilateral to the affected canal at the vHIT (No: canal not identified)

**VOR-gain for other ipsi vertical SC:** VOR-gain value for the other vertical canal ipsilateral to the affected canal at the vHIT (No: canal not identified)

**VOR-gain for contra SC coupled with affected SC:** VOR-gain value for the vertical canal of the unaffected side coupled to the affected canal at the vHIT (No: canal not identified)

**VOR-gain for contra HSC:** VOR-gain value for the horizontal semicircular canal of the unaffected side at the vHIT (No: canal not identified)

**VOR-gain for other contra vertical SC:** VOR-gain value for the other vertical canal of the unaffected side at the vHIT (No: canal not identified)

**cVEMPs ipsi**: cervical VEMPs detected over the sternocleidomastoid muscle ipsilaterally to the affected canal (n.p: not performed)

**cVEMPs contra**: cervical VEMPs detected over the sternocleidomastoid muscle contralaterally to the affected canal (n.p: not performed)

**oVEMPs ipsi**: ocular VEMPs detected under the eye contralaterally to the affected canal (n.p: not performed)

**oVEMPs contra:** ocular VEMPs detected under the eye ipsilaterally to the affected canal (n.p: not performed)
